# Loss of staminodes in *Aquilegia jonesii* reveals a fading stamen–staminode boundary

**DOI:** 10.1186/s13227-024-00225-3

**Published:** 2024-05-25

**Authors:** Jason W. Johns, Ya Min, Evangeline S. Ballerini, Elena M. Kramer, Scott A. Hodges

**Affiliations:** 1 Department of Ecology, Evolution, and Marine Biology, University of California Santa Barbara, Santa Barbara, CA 93106 USA; 2 Department of Ecology and Evolutionary Biology, University of Connecticut, 75 N. Eagleville Rd., Unit 3043, Storrs, CT 06269 USA; 3 Department of Biological Sciences, California State University Sacramento, 6000 J. St., Sacramento, 95819 CA USA; 4 Department of Organismic and Evolutionary Biology, Harvard University, 16 Divinity Ave., Cambridge, 02138 MA USA

**Keywords:** Columbine, Staminodia, Floral organ boundary, Floral organ loss, Quantitative trait locus

## Abstract

The modification of fertile stamens into sterile staminodes has occurred independently many times in the flowering plant lineage. In the genus *Aquilegia* (columbine) and its closest relatives, the two stamen whorls closest to the carpels have been converted to staminodes. In *Aquilegia*, the only genetic analyses of staminode development have been reverse genetic approaches revealing that B-class floral identity genes are involved. *A. jonesii*, the only species of columbine where staminodes have reverted to fertile stamens, allows us to explore the genetic architecture of staminode development using a forward genetic approach. We performed QTL analysis using an outcrossed F2 population between *A. jonesii* and a horticultural variety that makes fully developed staminodes, *A. coerulea* ‘Origami’. Our results reveal a polygenic basis for staminode loss where the two staminode whorls are under some level of independent control. We also discovered that staminode loss in *A. jonesii* is not complete, in which staminode-like traits sometimes occur in the inner fertile stamens, potentially representing a fading boundary of gene expression. The QTLs identified in this study provide a map to guide future reverse genetic and functional studies examining the genetic basis and evolutionary significance of this trait.

**Supplementary Information:**

The online version contains supplementary material available at 10.1186/s13227-024-00225-3.

## Background

Flowers are by far the most evolutionarily successful means of reproduction for plants [1]. Originally modified from leaves, subsequent modifications to every floral organ have produced a broad array of floral morphologies across angiosperms [2, 3]. Modifications to stamens have occurred in at least one species of ca. 30% of plant families [4, 5]. Often these modified stamens are rudimentary, but in some cases they maintain and/or gain some function [4]. The most common stamen modifications are ones that aid directly in pollination, such as becoming showy or developing a mechanical function like catapulting pollen onto a pollinator [4, 6]. Many stamen modifications result in their sterility, on a gradient from producing pollen without viable sperm to not producing anthers at all [5]. These modified stamens are referred to generally as staminodes (staminodia).

In the Ranunculaceae, a single clade encompassing the genera *Aquilegia*, *Semiaquilegia* and *Urophysa* [7–9], has sterile, elaborated staminodes, which are modified from the two stamen whorls positioned between the fertile stamens and fertile carpels [10–12]. These staminodes differ from the stamens by loss of anthers and modification of the filaments to become flat, laterally expanded, and ruffled. Occasionally, weakly chimeric organs develop, where a wild-type staminode has a reduced, apparently sterile anther at its apex [11, 13]. In *Aquilegia*, staminodes develop lignification on their adaxial epidermis and neighboring staminodes are often fused along their lateral edges, representing more extensive modification [13]. After anthesis, the outer floral organs senesce and abscise, yet the fused staminodes persist in a sheath around the developing carpels and are eventually sloughed off as the carpels expand [11, 14]. In addition, many genes associated with lignification, anti-herbivory and anti-microbial functions are upregulated in staminodes compared to the other floral whorls [11]. These features together suggest that staminodes may be adapted to provide defense against herbivory or microbial pathogens, although this has yet to be tested directly [10, 11, 13].

*Aquilegia jonesii* Parry, the only species of columbine lacking staminodes, presents a natural variant that we use here to investigate the genetic architecture of staminode loss, which may provide further insight into the molecular basis of staminode development [15]. *A. jonesii* is nested well within the *Aquilegia* clade indicating that staminodes were lost in this species [16, 17]. Despite this loss, no other obvious difference in organ identity, such as carpel number, distinguishes *A. jonesii* from other species of *Aquilegia,* suggesting that staminodes have reverted to stamen identity.

An obvious candidate gene for staminode loss would be an organ identity gene. In *Aquilegia*, three paralogs of MADS-box B-class floral identity genes are expressed during the early development of staminodes, *AqPISTILLATA* (*AqPI*), and two *APETALA3* paralogs (*AqAP3-1* and *AqAP3-2*) and each has been investigated in knockdown experiments with *A. coerulea* ‘Origami’ [11, 12, 18, 19]. Knockdown of *AqPI* results in the conversion of staminodes, stamens and petals to carpels [20] and knockdown of *AqAP3-1* results in the conversion of staminodes to carpels and/or chimeric carpel–stamen organs [12], making these genes unlikely to be completely responsible for staminode loss in *A. jonesii*. Knockdown of *AqAP3-2* results in the loss of anthers but has no obvious effect on staminodes [12]. Therefore, the maintenance of *AqAP3-2* expression in the inner stamen whorls of *A. jonesii* may cause the development of fertile stamens instead of staminodes.

Other possible candidate genes are those that have been found to be differentially expressed between stamen filaments and staminodes and involved with abaxial/adaxial polarity [11, 13]. In particular, using histology, RNAseq, and in situ hybridization in *Aquilegia*, Meaders et al. [13] found that staminodes differ from stamen filaments in the localization and expression levels of several developmental polarity genes known to be involved in laminar expansion, including the *YABBY* gene family transcription factors *AqCRABS CLAW* and *AqFILAMENTOUS FLOWER/YABBY1*. They also found upregulation in staminodes compared to stamens of two genes known to be involved in organ adhesion in *Arabidopsis*, *AqHOTHEAD* and *AqDEFECTIVE IN CUTICULAR RIDGES* [21, 22]. Modification of any of these loci could be involved in the *A. jonesii* phenotype.

In addition to or in concert with modifications to the genetic pathway specific to staminode development, a potential explanation for a loss of staminodes in *A. jonesii* could be its relatively few floral whorls. When comparing species of *Aquilegia* and close relatives, Tucker and Hodges [10] found a positive correlation between stamen and staminode number, where *A. ecalcarata* flowers with only 10 stamens (2 whorls) had only 1–3 staminodes, while those with 40–60 stamens made 10 staminodes (2 whorls). Similarly, the extremely small flowers of *Semiaquilegia adoxoides* produce just 8–14 stamens and 0–3 staminodes, while the larger flowered *S. guangxiensis* produces 20–30 stamens and ca. 10 staminodes [8]. Given that *A. jonesii* flowers are relatively small with fewer whorls of stamens than most other columbine species, we have also investigated whether the number of organ whorls is correlated with staminode production.

The natural variant *A. jonesii* provides an opportunity to use a forward genetic approach to further explore potential key genes in staminode development. We sought to determine if the loss of staminodes in *A. jonesii* has a simple genetic architecture, such as mapping to the *AqAP3-1/2* loci, whether it may involve any of the other associated genes described above, or if it involves other potentially novel pathways. To identify regions of the genome harboring causal genes, we used QTL analysis with an F2 population generated by crossing *A. jonesii* and a staminode producing variety of *Aquilegia*, *A. coerulea* ‘Origami’, henceforth referred to as ‘Origami’. In addition to using photographs of whole organs to identify the morphological nuances in the transition from stamens to staminodes, we also used histological analysis to characterize the cellular morphology of staminodes in the parents and the F2 population. Together, these analyses uncover more phenotypic and genotypic complexity for staminode development than previously described, and identify new candidate genes that could be involved in staminode development.

## Results

### Complexity and nuance in staminode phenotypes

**Fig. 1:**
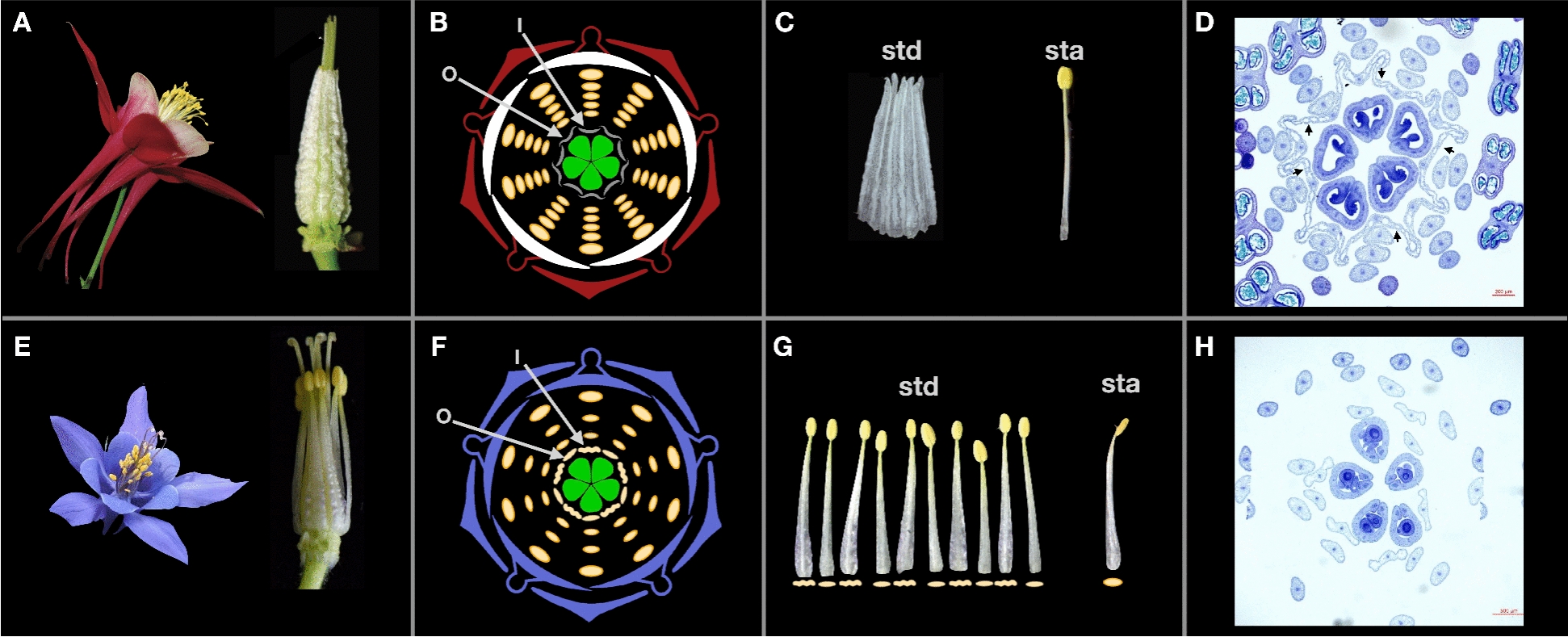
Floral phenotypes of ‘Origami’ (**A–D**) and *A. jonesii* (**E–H**). **A** and **E** Whole flowers (left) and after dissection to just the inner two staminode/stamen whorls and carpels (right). **B** and **F** Floral diagrams with the innermost whorl (I) and the next outer whorl (O) relative to the carpels indicated by arrows. For *A. jonesii*, morphological variation between the I-whorl (wavy symbol) and the O-whorl (flattened symbol) is depicted. **C** and **G** Dissected organs from the I and O whorls, labeled staminodia (std) on the left and a stamen (sta) on the right. Symbols under organs in **G** match those in **F**, depicting chimeric organs in *A. jonesii*. **D**, **H** Toluidine blue stained sections of young floral buds at stage 12 from Min & Kramer, 2017. **H** Image is from an F2 flower with the same phenotype as *A. jonesii*. Arrows indicate the inner whorl staminodes in panel D. Scale bars = 500 µm. Phenotypic scores for the *A. jonesii* flower are indicated in Additional file 1; Fig. S2 (AjBB3a). Phenotypic scores for the ‘Origami’ flower are all maximal staminode-like scores (Fig. 2)

‘Origami’ makes two whorls of well-developed staminodes that completely lack anthers and have flattened, laterally expanded, and ruffled filaments that fuse along their lateral margins to form a sheath around the carpels (Fig. 1A, B). There is a sharp boundary between the staminode whorls and the next outer whorl, which produces fully fertile stamens with anthers and narrow, round, unfused filaments (Fig. 1C, D). While *A. jonesii* has lost canonical staminodes, some staminode-like characteristics occasionally remain in the fertile stamens (Fig. 1E–H, Additional file 1; Figs. S1, S2). In the two innermost whorls of stamens, where staminodes normally develop in other *Aquilegia* species, the stamens make functional anthers but the filaments are often somewhat flattened and may have ruffled margins, increasingly so proximally, and to the greatest degree in the innermost whorl (Fig. 1E–H, Additional file 1; Figs. S1, S2). Furthermore, we observed that in the outer whorls of stamens, the filaments gradually become more narrow (i.e., typically stamen-like) without any lateral expansion (Additional file 1; Figs. S1, S2). Thus, the inner stamens of *A. jonesii* sometimes exhibit a degree of staminode-like traits. However, by far the largest phenotypic difference between ‘Origami’ and *A. jonesii* was in the two whorls closest to the carpels (Fig. 1C–D, G–H).

We used an outcrossed F2 population from a cross between *A. jonesii* and ‘Origami’ to map the genetic architecture of *A. jonesii*’s staminode loss. We phenotyped either one (62 plants) or two (169 plants) flowers per plant for a total of 400 flowers. Similar to *A. jonesii*, variation among F2 flowers revealed that the inner and outer whorls closest to the carpels sometimes differed from each other in their phenotypes, indicating variation in the boundary of organ identity (Additional file 1; Figs. S3, S4). Staminode or chimeric staminode–stamen organs were largely confined to these two whorls and therefore we restricted our analysis to them and phenotyped them separately (Additional file 1; Figs. S3, S4). Only 22 flowers from 19 plants had any amount of filament lateral expansion and ruffling outside these two whorls.

**Fig. 2:**
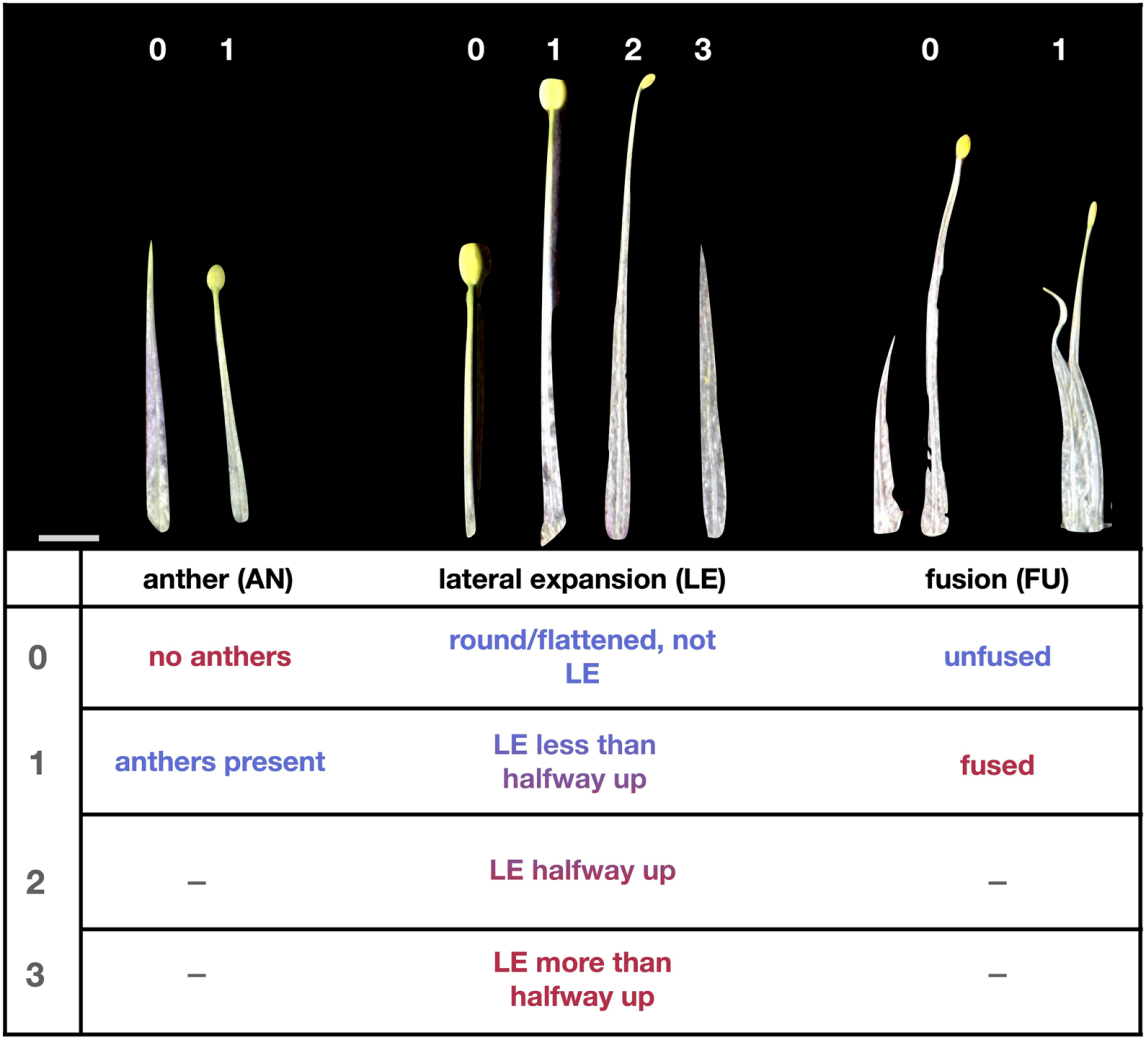
Examples of phenotype scores of subtraits. Organs were dissected from F2 plants representative of each phenotype score. Red text indicates an ‘Origami’-like (staminode-like) score and blue text indicates an *A. jonesii-*like (stamen-like) score. Intermediate colors are used for the intermediate LE scores of 1 and 2. Scale bar = 2 mm

Chimeric organs were complex and appeared to vary continuously across F2 plants. We chose to phenotype these organs using three subtraits: presence/absence of anthers (AN), lateral expansion of filaments (LE), and ± fusion of neighboring filaments (FU; Fig. 2). For instance, organs ranged from making no anther at all, to small, shriveled anthers, to pollen-producing anthers of various sizes (Additional file 1; Fig. S3). We did not measure the level of pollen viability of anthers, but focused our analysis on anther production of any kind with a binary score of presence/absence (Fig. 2). Filament lateral expansion was similarly continuous, varying from relatively round filaments to completely flattened and ruffled (Additional file 1; Figs. S3, S4). When the degree of LE varied within a filament, it always decreased from the proximal to the distal end. Given this variation in LE along the length of the filament, we scored LE with four ordered bins (Fig. 2, Additional file 1; Fig. S3): round or flattened, but not laterally expanded (0), flattened and laterally expanded less than halfway up the filament (1), flattened and laterally expanded halfway up the filament (2), and flattened and laterally expanded more than halfway up the filament (3). The degree of FU also varied somewhat continuously, but we scored the trait using a binary presence/absence of fusion (Fig. 2, Additional file 1; Fig. S3).

When two flowers from the same plant were measured they usually had identical phenotypic scores. For AN, 119 plants (70%) had the same score across both flowers in both the inner and outer whorls (Additional file 2; Table S1). Of the 50 plants with variation between flowers, 20 differed only in the inner whorl, 20 differed only in the outer whorl, and 10 differed in both whorls. Scores between flowers were also highly consistent for LE, where the same score was observed in 139 plants (82%) for the inner whorl and 123 plants (73%) for the outer whorl (Additional file 2; Table S2). Only four plants had flowers differing by a score of 3 in either whorl for LE. FU was the most consistent trait across flowers within a plant, where 154 plants (91%) had the same phenotype (Additional file 2; Table S3). Thus, for the QTL analysis, we averaged scores across plants with two flowers phenotyped.

Correlations were relatively high among subtraits using one flower from each of 231 plants (polychoric correlations; Additional file 2; Table S4). For correlation tests, we randomly chose one flower per plant to maintain the independence of each measurement. Mirroring the parental phenotypes, AN was negatively correlated with both FU and LE in both whorls (polychoric correlations ranged from − 0.57 to − 0.77), although these correlations were weaker than the positive correlations between LE and FU (0.88–0.95; Additional file 2; Table S4). The near perfect positive correlation of LE and FU is expected as FU can only occur with some amount of LE (Additional file 1; Figs. S3, S4; [13]).

The F2 plants varied in the degree of morphological difference between inner and outer whorls. We used one randomly chosen flower per plant for comparisons between whorls (the same flower as the one used for correlation tests). The majority of plants reflected the ‘Origami’ parent where the inner and outer whorls had the same phenotype (Additional file 2; Table S5). This was especially true for AN, where 72% had the same phenotype in both whorls and 28% produced anthers only in the outer whorl (Additional file 2; Table S5). LE was less consistent across whorls, where just over half (54%) of flowers had the same phenotype in both whorls and 46% had different phenotypes between whorls (Additional file 2; Table S6). Similar to *A. jonesii*, when the two whorls of a flower differed, the inner whorl was always more staminode-like (Additional file 2; Tables S5, S6). In addition, flowers with a strongly staminode-like inner whorl were more likely to have a staminode-like outer whorl (Additional file 2; Tables S5, S6).

Histological cross sections of one flower from each of ten F2 plants also revealed continuous variation in LE and FU, in addition to occasional variation between whorls (Additional file 1; Fig. S4). As sections were taken ca. halfway down the flower bud, AN phenotypes were not visible. The outer whorl, in line with the carpels, was always more stamen-like (Additional file 1; Fig. S4).

While the number of sepals, petals, and carpels is almost always five across both parents, ‘Origami’ flowers have many more stamens (x̅ = 60.6, s = 5.6, *n* = 44; not counting the two whorls of staminodes) than *A. jonesii* flowers (x̅ = 34.2, s = 4.9, *n* = 6; t = − 16.4, *p* << 0.001; Additional file 1; Fig. S5). Thus, we sought to determine if floral organ number (FON) is correlated with staminode production, and if this varies for the inner versus outer whorl. Logistic regression revealed that FON was significantly correlated [odds ratio (OR) = 0.97, *p* = 0.001, ɑ = 0.05] with all three subtraits, though given that the ORs are all near 1.0, the effect of FON is very small (Additional file 1; Fig. S5 and Additional file 2, Table S7). Whorl identity had a much larger effect on AN and LE than FON, where the outer whorl was more likely to make anthers [OR (outer whorl): 2.83, *p* < 0.001, ɑ = 0.05] and was less laterally expanded [OR (outer whorl): 0.35, *p* < 0.001; Additional file 1, Fig. S5A, B and Additional file 2, Table S7]. The interaction term between FON and whorl was not included in the final models for AN or LE as it was not significant. Whorl was not included in the FU model, as fusion occurs between whorls, and thus cannot differ between them. While there were significant correlations, there was nearly complete overlap in trait values among FON counts (Additional file 1; Fig. S5) indicating that little variation in staminode development is explained from FON (Additional file 2; Table S7).

### F2 genotype data

Using whole genome skim sequencing, we identified 849,724 SNPs across 599 marker bins (see Methods) spanning the genome that were useful for genotyping and genetic map construction. Of the 334 plants, 5 did not have enough coverage to genotype, leaving 329 individuals to assemble a genetic map. The genetic map had similar ordering of marker bins to their physical order in the *A. coerulea* ‘Goldsmith’ V3 reference genome [23]. However, there was some discordance between the physical and genetic marker positions, mostly in pericentromeric regions, similar to the findings in other crosses of *Aquilegia* (Additional file 1; Fig. S6; [24–27]. Recombination rates were higher toward the ends of chromosomes (Additional file 1; Fig. S6). Patterns of transmission distortion varied across the seven chromosomes, with distortion favoring the ‘Origami’ allele in some regions and the *A. jonesii* allele in others (Additional file 1; Fig. S7).

### Polygenic basis of staminode loss

**Fig. 3:**
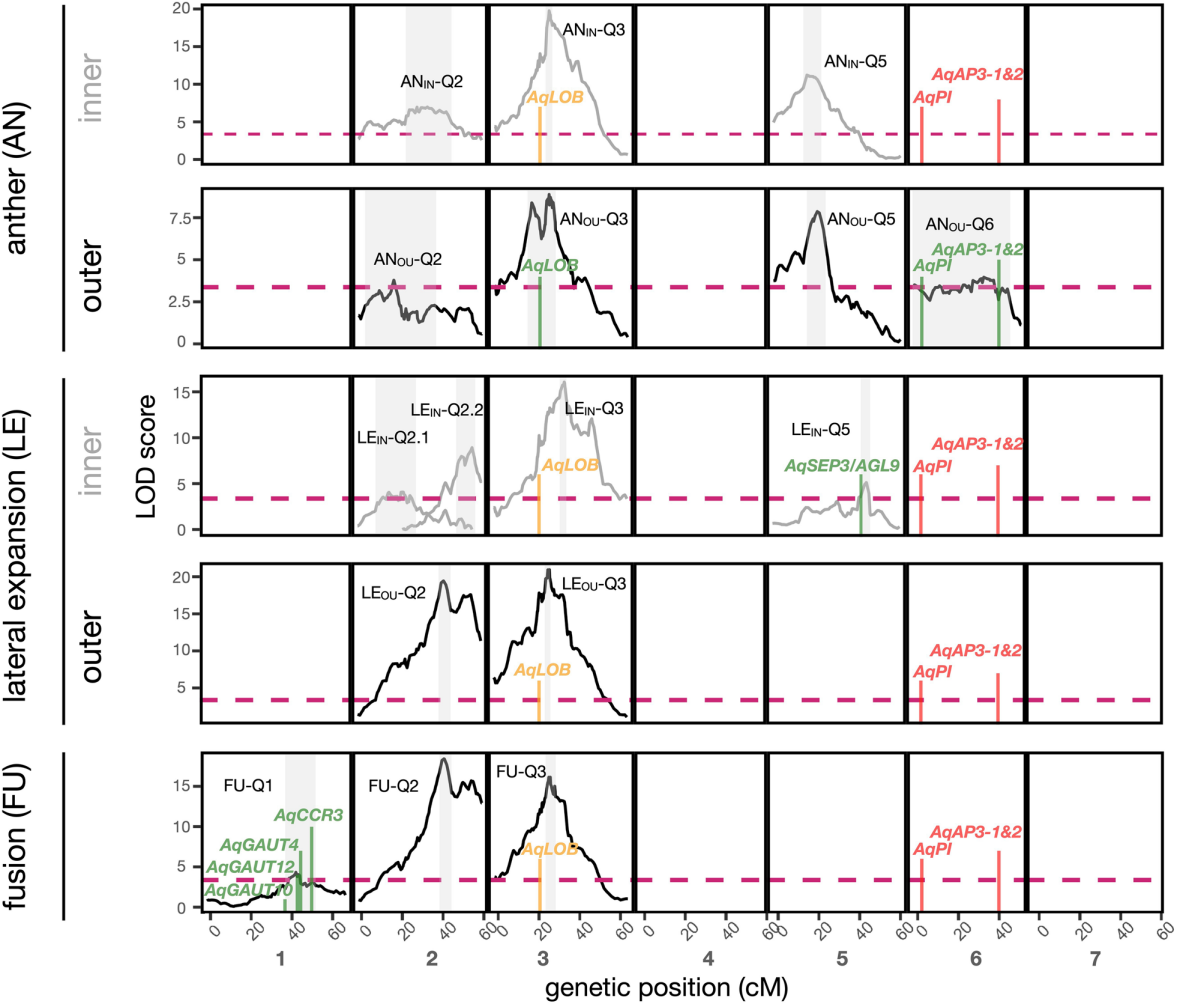
QTL maps for each subtrait that differentiate stamen and staminode identity for the inner and outer whorls of *A. coerulea* ‘Origami’ and *A. jonesii,* across the 7 *Aquilegia* chromosomes. LOD (logarithm of odds) profiles reflect the best multiple QTL model associated with each subtrait and whorl, with those affecting the inner whorl in grey and the outer whorl, or both whorls in black. LOD profiles are only shown for chromosomes where significance is reached and peak QTL are labeled by subtrait, whorl if applicable, and chromosome number. Shaded areas represent 1.5 LOD intervals for each QTL peak. Dashed horizontal line represents the LOD significance threshold of 3.37. A single QTL map for FU is shown since it combines organs from both the inner and outer whorls. Positions of potential candidate genes are indicated using vertical lines, with those falling within a 1.5 LOD interval (green), those outside a 1.5 LOD intervals but within a LOD profile above the significance threshold (orange) and B-class floral identity genes falling in a region with a LOD profile below the significance threshold (red). *AqLOB: LATERAL ORGAN BOUNDARIES, AqPI: PISTILLATA, AqAP3-1&2: APETALA3-1&2, AqSEP3: SEPALLATA3, AqAGL9: AGAMOUS-LIKE9, AqCCR3: CRINKLY-RELATED3, AqGAUT4,10,12: GALACTURONOSYLTRANSFERASE4,10,12*

Overall, we identified 9 independent QTL (non-overlapping 1.5 LOD intervals) across the three subtraits and two whorls (Table 1 and Fig. 3). Full models explained 44–53% of the total percent variation explained (PVE). We found one ‘major effect’ QTL, defined as having greater than 25% PVE, for LE in the outer whorl on Chr3 (LE_OU_-Q3, 25.5 PVE). Multiple QTL were identified for each subtrait, many of which had overlapping 1.5 LOD intervals both for a single subtrait across the two whorls as well as across subtraits within a whorl, suggesting pleiotropic effects for genes within these QTL. For instance, the 1.5 LOD intervals overlap among QTL for nearly all three subtraits across both whorls on Chr3 (excluding LE_IN_-Q3). Similarly, the intervals for a Chr2 QTL overlap across most subtraits in both whorls (excluding AN_OU_-Q2 & LE_IN_2.1&2.2). We did not detect any significant interactions between QTL. As FON never explained more than 3% of the variation, it was not included as a covariate in final QTL models.

**Table 1:** QTL results for each subtrait and whorl

Subtrait	Chr	Name	1.5 LOD int (cM)	1.5LOD marker	Peak position (cM)	LOD	PVE	Total PVE	Additive effect	Dominance deviation	Degree of dominance
Anther, inner	2	AN_IN_-Q2	22.7–44.6	2.52–2.74	33	7	7.3	50.8	− 0.19	− 0.04	0.21
	3	AN_IN_-Q3	24.4–27.6	3.093–3.116	26.1	19.8	23.8		− 0.29	− 0.19	0.66
	5	AN_IN_-Q5	14.3–22.9	5.1–5.17	16	11.2	12.3		− 0.23	0.02	0.09
Anther, outer	2	AN_OU_-Q2	3.2–37.2	2.05–2.69	16.9	3.8	4.4	44.3	− 0.13	0.04	0.31
	3	AN_OU_-Q3	15.9–29.2	3.023–3.125	26.1	8.9	10.8		− 0.22	0.02	0.09
	5	AN_OU_-Q5	16.0–25.0	5.12–5.18	21.9	7.9	9.5		− 0.15	− 0.11	0.73
	6	AN_OU_-Q6	0.0–46.9	6.01–6.44	34.2	4	4.6		− 0.14	− 0.07	0.5
Lateral expansion, inner	2	LE_IN_-Q2.1	8.6–28.0	2.09–2.61	21.4	4.1	4	53	0.27	0.22	0.81
	2	LE_IN_-Q2.2	47.2–56.3	2.76–2.84	54.8	8.9	9.2		0.39	0.21	0.54
	3	LE_IN_-Q3	31.7–34.8	3.126–3.129	33.8	16.1	17.8		0.47	0.34	0.72
	5	LE_IN_-Q5	42.2–46.7	5.58–5.63	44.9	5.1	5.1		0.55	0.45	0.82
Lateral expansion, outer	2	LE_OU_-Q2	38.8–44.6	2.7–2.74	41.2	19.4	23.3	50.8	0.79	0.39	0.49
	3	LE_OU_-Q3	24.4–27.1	3.093–3.08	26.7	21	25.5		0.87	0.3	0.34
Fusion	1	FU-Q1	37.2–51.8	1.54–1.69	42.3	4.3	4.4	50.7	− 0.16	0.02	0.13
	2	FU-Q2	38.8–44.6	2.7–2.74	40.9	18.4	21.9		0.29	0.16	0.55
	3	FU-Q3	24.4–29.2	3.093–3.125	26.7	16.1	18.7		0.29	0.09	0.31

“inner” and “outer” refer to the staminode whorls as indicated in Fig. 1

chr, chromosome; 1.5 LOD int, 1.5 LOD interval; PVE, Percent Variance Explained. Additive effects are in terms of the ‘Origami’ all

For all QTL except one, we observed parental divergence where F2 plants with alleles for a given parent were more likely to make organs with the same phenotype as the parent (e.g., for *A. jonesii*, AN = 1, FU = 0, LE = 0; Table 1, Additional file 1; Fig. S8–S10). The only exception was FU-Q1, where individuals homozygous for the *A. jonesii* allele were more likely to be fused than individuals heterozygous or homozygous for the ‘Origami’ allele, however this is a minor effect QTL, defined as having less than 10 PVE, with 4.4 PVE (Table 1, Additional file 1; Fig. S10). Most traits showed additive patterns of inheritance for the parental alleles, although ‘Origami’ alleles were dominant slightly more often than *A. jonesii* alleles (Table 1; Additional file 1; Fig. S8–S10).

#### Anther (AN)

The best fit model for AN identified three QTL for the inner whorl (AN_IN_) and four QTL for the outer whorl (AN_OU_; Table 1, Fig. 3), with 51 and 44 total PVE, respectively. Across the three QTL common to both whorls, AN_IN_ had higher LOD scores than AN_OU_. Among the subtraits, AN had the most consistent QTL across the two whorls, with their 1.5 LOD intervals overlapping on chromosomes 2, 3 and 5. Q3 explained the most variation for both AN_IN_ (24%) and AN_OU_ (11%). A minor effect QTL for AN_OU_ was detected on Chr6 (4.6 PVE). While *AqAP3-1&2* are within AN_OU_-Q6, it is of minor effect and the 1.5 LOD interval covers most of the chromosome, indicating these genes play a minor role, if any, in *A. jonesii*’s gain of anthers. Q5 does not overlap across AN and LE indicating that it does not have pleiotropic effects across subtraits. *A. jonesii* alleles for AN QTL always promoted anther presence (Table 1, Additional file 1; Fig. S8–S10). FON only explained 0.9% of the variance, suggesting it plays a minimal role in this subtrait, and was not included in the final QTL model.

We focused our candidate gene search for AN on homologs of genes and gene families implicated in anther development, the establishment of ab-adaxial polarity, and organ boundaries. We did not find any genes known to be involved in anther development or ab-adaxial polarity within the 1.5 LOD intervals of all QTL (henceforth referred to as “within QTL”). We did find a homolog of a gene involved in the establishment of organ boundaries in plants, *LATERAL ORGAN BOUNDARIES* (*LOB*), which was within AN_OU_-Q3 and just outside AN_IN_-Q3 (Fig. 3; Additional file 2; Table S8). *AqPI*, *AqAP3-1*, and *AqAP3-2* all fell within AN_OU_-Q6, although it was of minor effect and the 1.5 LOD interval covered 90% (in cM) of the chromosome (Fig. 3, Table 1, Additional file 2; Table S8).

#### Lateral expansion (LE)

The best fit models for LE revealed four QTL for the inner whorl (53% PVE) and 2 QTL for the outer whorl (51% PVE; Table 1, Fig. 3). For both whorls, the largest effect QTL was on Chr3, although the 1.5 LOD regions did not overlap between whorls (Table 1, Fig. 3). We detected two QTL for LE_IN_ and one QTL for LE_OU_ on Chr2. ‘Origami’ alleles always produced more flattened and laterally expanded organs (Table 1, Additional file 1; Fig. S9). FON only accounted for 1.6 PVE in LE_IN_ and 1.9 PVE in LE_OU_, and was not included in the final QTL models.

For LE-specific candidate genes, we considered homologs of genes and gene families previously implicated in floral organ identity, ab-adaxial polarity, organ boundaries, and laminar expansion of stamens (Additional file 2; Table S8). The only notable gene we found was *AqSEPALLATA3/AGAMOUS-LIKE9 (AqSEP3/AGL9)*, which fell within LE_IN_-Q5, unique to LE for the inner whorl (Fig. 3, Table 1, Additional file 2; Table S8). None of the differentially expressed genes in Meaders et al. [13] associated with expansion of lateral organs fell within LE QTL.

#### Fusion (FU)

The best fit model for FU identified three significant QTL, which explain 51% of the variance (Table 1, Fig. 3). The LOD profiles of FU-Q2 and FU-Q3 were nearly identical to that of LE_OU_-Q2 and LE_OU_-Q3. We detected moderate effect QTL, defined as having 10–25 PVE, on Chr2 and Chr3, where FU-Q2 had the largest effect (22 PVE). Although FU-Q1 was narrowly significant, explaining an estimated 4.9% of the variance, it was unique to FU, suggesting it may harbor genes specific to organ adhesion. However, FU-Q1 was the only QTL for FU where plants with ‘Origami’ alleles were less likely to be fused (Table 1, Additional file 1; Fig. S10). FON explained more variation for FU than for AN or LE, however it was marginal (3 PVE), so it was not included in the final QTL model.

We searched for genes and gene families known to be involved with fusion and organ adhesion in other taxa and found candidates for staminode fusion in *Aquilegia* (Additional file 2; Table S8) within FU QTL. Of particular interest were genes within FU-Q1, as this QTL was unique to FU. In addition, this was the only QTL within which we found FU-specific candidates, including a paralog of *CR4, CRINKLY-RELATED3* (*CCR3*), and three galacturonosyltransferase homologs (*GAUT4, 10, & 12*; Fig. 3, Additional file 2; Table S8).

## Discussion

Using *Aquilegia jonesii* in a QTL experiment to dissect the genetic basis of staminode development, we found a complex pattern of morphological variation among the F2 population involving multiple QTL, with some QTL affecting specific subtraits of staminode morphology (Table 1, Fig. 3). We found that the two whorls comprising normal staminode development can differ phenotypically and while the whorls have largely overlapping QTL, they also have whorl-specific QTL. Notably, none of the previously identified candidate genes for staminode development using reverse genetics were within QTL. This new understanding of the complex genetic architecture for staminode development will guide future reverse genetic approaches. Finally, we found that while *A. jonesii* has functionally lost staminodes, some of its stamens retain remnants of staminode morphology which display a gradual boundary beyond the normal position of staminodes (Fig. 1, Additional file 1; Figs. S1, S2). Thus, it appears that staminode development, and its loss in *A. jonesii,* involves multiple genetic factors controlling identity and organ boundaries.

### Fading boundary between stamens and staminodes in *A. jonesii*

Upon close examination, we found that *A. jonesii*’s loss of staminodes is not absolute. Instead, there is often some amount of flattening and lateral expansion of stamen filaments, decreasing in a gradient outward from the carpels (Additional file 1; Figs. S1, S2). The degree of this gradient varied among the flowers we examined, but the strongest difference was always between the “inner” and “outer” whorls where staminodes normally develop in other species (Fig. 1, Additional file 1; Figs. S1, S2). Within F2 plants, stamens with flattening and lateral expansion were almost always restricted to the two whorls where staminodes normally develop (Additional file 1; Fig. S3), suggesting that the *A. jonesii* parent may have a cryptic boundary between the typical staminode and stamen whorls. Although F2 plants generally did not have the same extent of fading boundary into the outer stamens as observed in some *A. jonesii* (Additional file 1; Fig. S3), the leaky stamen–staminode boundary observed in the F2 between the “inner” and “outer” whorls allowed us to differentiate the genetic architectures between them.

This gradient in staminode organ identity and incomplete loss of staminodes in *A. jonesii* contrasts with the floral homeotic mutants found in populations of *A. coerulea*, which exhibit a complete loss of petals [28–30]. *A. jonesii*’s gradient is more reminiscent of the “fading boundaries” observed in many basal angiosperms, which has been attributed to gradients in B-class organ identity gene expression across whorls [31–35]. While *A. jonesii*’s gradient differs from basal angiosperms in that it only involves stamen whorls, it may be similarly attributed to gradients in the expression of B-class genes such as *AqAP3-1*. Interestingly, our QTL analysis reveals that *A. jonesii*’s staminode loss is due to mutations in genes with up- and/or downstream activity of the B-class genes rather than to the B-class genes themselves.

### Organ loss via changes to multiple genes of minor/moderate effect

Changes to a single or few early acting developmental genes can lead to the loss or conversion of an organ, even when the organ is relatively complex [28, 36–38]. However, we found multiple QTL for each subtrait and only one had a marginally large (> 25% PVE) effect (Table 1, Fig. 3), suggesting that the loss of staminodes in *A. jonesii* was likely polygenic and complex. QTL associated with multiple subtraits may hold genes with epistatic effects, such that the activity of subtrait-specific genes (e.g., genes responsible for pollen development) depend on the upstream activity of genes which establish the stamen/staminode boundary. Additionally, or alternatively, genes in these QTL may interact with floral identity genes (i.e., B-class transcription factors) upstream of subtrait-specific genes. Detecting a significant epistatic interaction between QTL requires a strong interaction due to the additional degree of freedom for the interaction term [39]. Thus, while we did not find any significant QTL x QTL interactions in this study, future studies with a larger sample size should not discount the possibility that QTL common to multiple subtraits act epistatically to subtrait-specific QTL.

Notably, when QTL for a trait across both whorls overlapped, the effect sizes varied between the inner and outer whorls. For AN, the inner whorl had consistently larger LOD scores and narrower 1.5 LOD intervals (Table 1, Fig. 3), suggesting that the genes responsible for anther development in *A. jonesii’s* two whorls closest to the carpels have a greater effect once the stamen/staminode boundary is established. In contrast for LE, QTL had consistently larger and narrower LOD scores in the outer whorl (Table 1, Fig. 3), suggesting that genes important for LE may have a greater effect before or during the establishment of the stamen/staminode boundary. Notably, for each of these traits, the whorl with fewer QTL in its respective multiple QTL model also had greater PVE, which inherently inflates the PVE of each QTL compared to models with more QTL terms [40]. However, when we only included the two QTL with the highest PVE for AN and LE in both whorls, the differences in effect sizes between whorls remained. Thus, while genes under the majority of QTL appear to affect staminode development in both whorls, the degree of their effect depends on where the boundary between stamens and staminodes is established.

As the Chr5 QTL is unique among subtraits to both whorls of AN (Table 1, Fig. 3), this region is likely to harbor genes specific to anther development. We did not identify any genes previously implicated in anther development or ab-adaxial polarity establishment within this QTL, suggesting it may hold gene(s) with functions upstream of anther development itself and/or anther development genes that have yet to be discovered in model taxa.

During early floral meristem development in *Aquilegia*, anthers appear before filaments [10, 41]. Thus, the genetic pathways affecting *A. jonesii*’s retention of anthers in the staminode whorls could be at least partially epistatic to processes affecting filament morphology in the LE and FU subtraits. We identified *AqLATERAL ORGAN BOUNDARIES* (*AqLOB*) as a plausible candidate for stamen/staminode boundary establishment, as it was within the largest QTL for AN_OU_ on Chr3 (Table 1, Fig. 3, Additional file 2; Table S8). While *LOB* has mostly been directly implicated in vegetative organ boundary establishment, it is expressed in early floral buds in *A. thaliana*, suggesting it may play a role in floral organ boundary establishment [42–44]. Additionally, ectopic *LOB* expression in *A. thaliana* causes misshapen, chimeric organs and sterility [42]. As *AqLOB* is also expressed in early floral buds in *Aquilegia* [45], it could be involved in establishing the transition between stamens and staminodes.

Although there can be substantial variation between whorls in the extent of LE, we would expect LE in both whorls to be controlled by the same genes. Surprisingly, there were inconsistencies across whorls in QTL maps for LE, where none of the 1.5 LOD intervals for any QTL overlapped (Table 1 and Fig. 3). Therefore, it appears that the morphology of these two whorls is at least partially influenced by different genes. Flowers on the same F2 plant were more likely to differ in LE_OU_ than LE_IN_ (Additional file 2; Table S2), suggesting that there may be more of an environmental and/or stochastic effect on LE in the outer whorl. The presence of both more variation and larger and narrower LOD intervals for QTL in the outer whorl seems contradictory, as we would expect more non-genetic variation to weaken QTL. However, this would make biological sense if genes that positively regulate staminode identity and/or negatively regulate stamen identity have a greater effect before the stamen/staminode boundary is established. In the context of a fading boundary with a gradient of stamen/staminode gene expression, there is likely more competition with stamen identity genes in the outer whorl, and thus more phenotypic variation.

The QTL on Chr5 (LE_IN_-Q5) was unique to LE (non-overlapping with AN) for the inner whorl, and includes *AqSEP3* (Table 1, Fig. 3, Additional file 2; Table S8). *SEPALLATA* genes, which are E-class genes in the canonical ABCE model of floral development, have undergone lineage-specific duplication and diversification in the Ranunculaceae [46]. In *Arabidopsis*, *SEPALLATA3/AGAMOUS-LIKE9* (*SEP3/AGL9*) forms a heterotetramer complex with *AG*, *AP3*, and *PI* (*PISTILLATA*) to control stamen development. While *SEP3* appears to be largely involved in gynoecium development in *Thalictrum thalictroides* (Ranunculaceae), its knockdown caused chimeric organs across all three floral organs (*Thalictrum* lacks petals; [46]). Given *SEP3*’s involvement in floral development and its occurrence under LE_IN_-Q5, it may be involved in causing lateral expansion in *Aquilegia* staminode filaments.

The LOD profiles for FU and LE_OU_ were nearly identical (Table 1, Fig. 3), which was expected given the near perfect correlation values between the two traits (Additional file 2; Table S4). Thus, it is difficult to conclude from these data whether FU is under unique genetic control or whether it is a spatial result of the laterally expansion and bending patterns of neighboring organs. As the degree of FU varies across *Aquilegia* species [13], this could be further examined by crossing columbine species that make fused and unfused staminodes to identify loci responsible for FU itself. A notable difference between FU and LE is the Chr1 QTL unique to FU (Table 1, Fig. 3), and genes within this region may harbor candidates for FU-specific processes.

Fusion of neighboring staminodes involves adhesion of epidermal cells, which is a process often mediated by interactions between pectin molecules within the cuticle [47]. Homogalacturonan (HG), which is polymerized by galacturonosyltransferases (*GAUT*s), is an abundant pectin molecule in plant cell walls. Given the direct involvement of *GAUT* genes in pectin biosynthesis and their previous implication in cell–cell adhesion [48], the three *GAUT* homologs found within the 1.5 LOD interval of FU-Q1 are interesting candidates for staminode fusion (Fig. 3, Additional file 2; Table S8). Finally, although *CCR3* has not been directly implicated in organ fusion to our knowledge, it is a paralog of *CRINKLY4 (CR4)*, which is involved in cuticle formation and *cr4* mutants have abnormal fusion of epidermal cells [49]. Together, these homologs of cuticle biosynthesis genes under the Chr1 QTL unique to FU are promising candidates for a genetic pathway associated with the fusion of neighboring staminodes in *Aquilegia*.

### Variation in B-class genes is not responsible for staminode loss

Previously, *AqAP3-1* and *AqPI* were the only genes shown to have a functional effect on staminode identity, with *AqAP3-1* apparently neofunctionalized to promote staminode identity and *AqAP3-2* necessary for anther development [12, 19]. Thus, we initially hypothesized that *A. jonesii*’s loss of staminodes was due to variation in one of these B-class genes. However, while both of these genes colocalize with AN_OU_-Q6, this QTL is of minor effect and covers 90% of the chromosome, providing weak evidence for their involvement in *A. jonesii*’s staminode loss (Fig. 3, Additional file 2; Table S8). If changes to any of these genes do play a role, it is minor compared to genes within larger QTL that likely function up or downstream of B-class floral identity genes.

Importantly, although mutations in *A. jonesii*’s B-class genes apparently did not lead to staminode loss, this does not negate their involvement in staminode development. Our results may be partially explained by trans-acting factors that affect B-class gene expression. Thus, it would be informative to compare the expression profiles of *AqAP3-1*, *AqAP3-2*, and *AqPI* in early developing flowers with and without staminodes. Differential expression patterns of these B-class genes between staminode-position phenotypes would further implicate their role in determining stamen/staminode identity, and may inform whether genes underlying QTL act up or downstream of the B-class genes. Unfortunately, expression analysis in this case is extremely difficult because *A. jonesii* has single-flowered inflorescences that are large and well-developed by the time they begin to emerge from the plant crown, precluding the analysis of very early developmental stages. Most other columbines have inflorescences with many flowers that facilitate examination of very early developmental stages. One possible future approach would be to use F2 individuals that differ in staminode phenotypes, but also have multi-flowered inflorescences allowing the dissection of early developmental stages.

### Floral organ number plays little to no part in *A. jonesii*’s staminode loss

As noted previously, positive correlations between stamen and staminode numbers have suggested a potential role for the number of organ whorls and staminode production [10, 27]. However, we found only a very minor potential role for FON on staminode production with less than 3 PVE in our multiple QTL analyses. It is notable that there are species of *Aquilegia* with smaller flowers than *A. jonesii* that also make fully developed staminodes such as *A. saximontana* and *A. laramiensis*. Thus, while there may be a role of FON on staminode production in some species, FON can be largely decoupled with staminode organ identity in *A. jonesii*.

## Conclusion

The loss of staminodes in *A. jonesii* could have been caused by mutation to a master organ boundary establishment or organ identity gene. However, our study reveals a polygenic basis for the loss of staminodes in *Aquilegia jonesii* and thus the likely complex origin of staminodes. Additionally, closer observation of staminode organs in *A. jonesii* revealed important caveats to its loss, uncovering a previously unrealized porosity between the stamen/staminode boundary in *Aquilegia*. Further investigation of this dynamic in *Aquilegia* and other taxa with chimeric organs will bolster our understanding of the evolution of novel organs, their developmental boundaries, and how these characters may influence fitness.

## Methods

### Crossing experiment

Pollen from an *Aquilegia jonesii* plant collected from a wild population (Hunt Mtn Rd, WY) was used as the paternal parent and an *A. coerulea* ‘Origami’ plant as the maternal parent to produce the F1 generation. This F1 plant was self-pollinated to produce an F2 population. F1 and F2 seeds were soaked in 100mM gibberellic acid overnight prior to planting to induce germination.

Plants were germinated and grown in a custom soil mix (4 parts pumice, 2 parts perlite, 2 parts Foxfarm™ potting soil, 1 part vermiculite, 0.5 parts sand, 0.01 parts fertilizer) at 18 ºC with a 12/12 light schedule. Seeds were germinated in individual 2” pots then grown to a point where we determined that they were strong enough to transfer to 4” pots. Plants were further grown to near reproductive maturity and transferred to 1 gallon pots. To induce flowering, all F2 individuals were vernalized at 4 ºC for ca. 12 weeks.

### Phenotyping

We phenotyped flowers by first removing the petals and sepals, counting stamens as they were removed, and scoring staminodes as we removed them. All three subtraits were more conducive to qualitative measurements than quantitative. If organs came off of the flower together when dissected, they were considered fused (Fig. 2, Additional file 1; Fig. S3). Four photographs were taken of each flower: whole, petals and sepals removed, stamens removed, and staminodes removed. Phenotypes were later confirmed from photographs. Phenotypes for a given whorl were assigned based on the phenotypes of 3/5 organs in that whorl. When two flowers were measured for a plant, we averaged the values of each subtrait, which is why some plants have non-integer values. We used polychoric correlation tests, which are used when phenotypes are categorical and ordered.

### Genotyping

Genomic DNA (gDNA) was isolated from young leaves of all F2 individuals with a lab-based method using Qiagen reagents and the BioSprint 96 (Qiagen; see [28]). Genomic DNA quantities were verified via Qubit. Whole genome libraries were prepared using 1/2 reactions with the iGenomx Riptide 96-plex kit (now TWIST biosciences), analyzed on a Bioanalyzer at the UCSB Bio-Nanostructures Laboratory, multiplexed, then sequenced (~ 1x) at UC Davis DNA Technologies Core on one lane of a Novaseq S6 with 150PE reads. Sequence data for the *A. jonesii* parent were generated on an earlier run, where gDNA was isolated via the above method, a library was prepared using a 1/2 reactions of the NEBNext Ultra II DNA Library Prep Kit for Illumina (New England Biolabs), and was sequenced with other samples on a Novaseq S6 at the UC Davis DNA Technologies Core.

Sequencing data were demultiplexed by the UC Davis DNA Technologies Core, then paired-end reads were aligned to the *Aquilegia coerulea ‘*Goldsmith’ V3 reference genome using the Burrows-Wheeler Aligner (see [23] for parameters and details). F2 sequence data were aggregated to create a.bam file of the F1 parent. SNPs were called across this file using Samtools (v1.8) mpileup with options -q 30 -B -u -v -f and vcf files were created with bcftools (v1.10.1) call with options -vm -f GQ (see [24] for genotyping details). As we only had deep-coverage sequence for the *A. jonesii* parent we used the 1,039,650 SNPs heterozygous in the F1 and homozygous in the *A. jonesii* parent to genotype F2s. We called SNPs across these usable sites for each F2 using the above method. Allele frequencies for each F2 were calculated across 693 marker bins ranging from 50 kb to 1 Mb using custom scripts (see [24] for custom scripts). We used smaller bin sizes in regions of higher recombination (chromosome ends) and in areas where there was discordance between the physical and genetic maps (see below).

### Genetic map

We made a genetic map using the R/qtl package [40]. In several regions across our genetic map, there were particular markers that had a recombination event in almost all individuals. Similar to previous crosses using *Aquilegia* [24, 26, 27], we determined that this was due to either mis-assemblies in the reference genome or structural differences between the reference genome and the taxa in our cross. Thus, we manually reconstructed our genetic map, moving markers according to parsimony.

### QTL analysis

QTL analysis was performed using the R/qtl package [40]. We first ran the ‘scanone’ analysis to independently test whether each marker has a QTL and identify general regions of the genome associated with the trait. We then ran ‘scantwo’, which looks at each possible pair of markers and determines whether each of them holds a QTL. The significance cutoffs for QTL were determined by 1000 random permutations of the data to determine the highest LOD score produced by random chance. Using the results of ‘scanone’ and ‘scantwo’, we used ‘makeqtl’ to predict the best model, then ‘refineqtl’ to determine the most likely locations for each QTL given the model as a whole, and finally ‘fitqtl’ to run the model. The model that explained the most variation and had the highest overall LOD score was chosen. Any loci that did not increase the overall LOD score by more than the significance threshold were excluded from the final model.

### Candidate gene analysis

Genes were searched in the ‘Araport11’ *Arabidopsis* genome by keyword using Phytozome ([50, 51]; https://phytozome.jgi.doe.gov) and compared to the *Aquilegia* proteome using the ‘Protein Homologs’ tool. Results were then filtered based on sequence similarity and alignment length, and reciprocal best hits were identified as homologs.

### Histology

Developing flower buds (1–3 cm) were harvested, photographed, measured, and dissected from 10 individuals representing an array of staminode phenotypes and genotypes at the various QTLs. See [13] for histology protocol.

### Supplementary Information

Supplementary Material 1. Contains 10 .pdf figures supplemental to the results of the main text.

Supplementary Material 2. Contains 8 .pdf tables supplemental to the results of the main text.

Supplementary Material 3. staminode_f2_phenotypes.csv: phenotypic data for F2 plants.

Supplementary Material 4. staminode_jon_ori_phenotypes.csv: phenotypic data for jonesii and origami plants.

## Data Availability

Sequence data that support the findings of this study have been deposited in the National Institute of Health (NHI) Sequence Read Archive (SRA) with BioProject accession number PRJNA1089234. See additional files for phenotypic data. All code is available in Jason Johns’ Github repository (jasonwjohns/aquilegia_staminodes).
