# Supplementary material for: Loss of staminodes in Aquilegia jonesii reveals a fading stamen–staminode boundary
Source: EvoDevo. 2024 May 25;15:6. doi: 10.1186/s13227-024-00225-3 (PMC11127400; doi:10.1186/s13227-024-00225-3)
Supplement: Supplementary file 1 — Supplementary Material 1. Contains 10 .pdf figures supplemental to the results of the main text. [file 13227_2024_225_MOESM1_ESM.pdf]

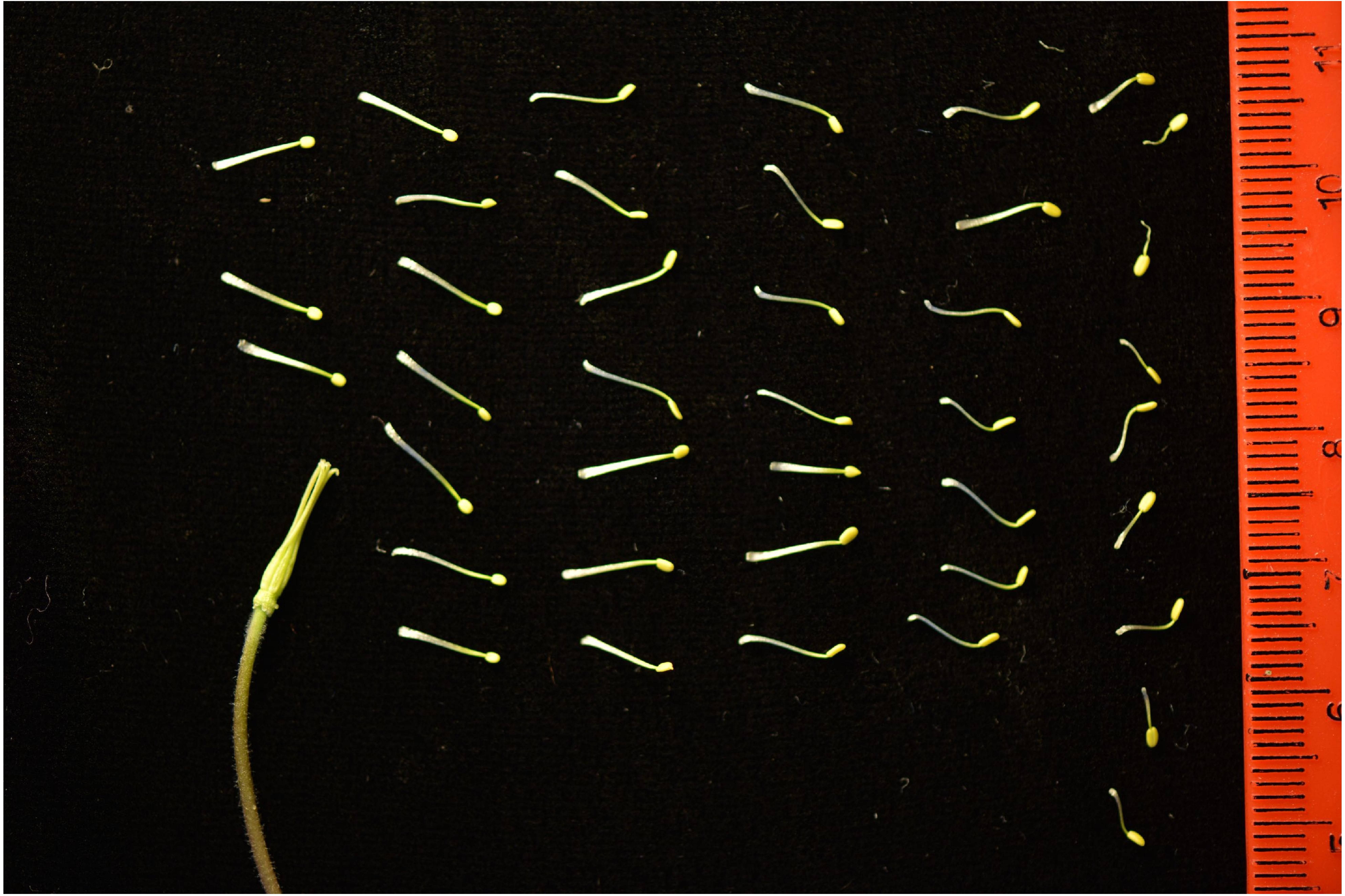

Figure S1. Photograph of an *A. jonesii* flower with all stamen whorls dissected and the carpels attached to the pedicel. Stamens are placed from inner (L) to outer (R). Filaments show some flattening, lateral expansion, and ruffling toward the base, especially in inner whorls. The flower was dissected from a greenhouse grown plant grown from seed collected from Hunt Mnt. Rd., the same population as the pollen parent. Scale is mm.

plant	selection of organs across all stamen whorls	staminode whorls	anther		lateral expansion		fusion
			inner	outer	inner	outer	-
AjDM4b	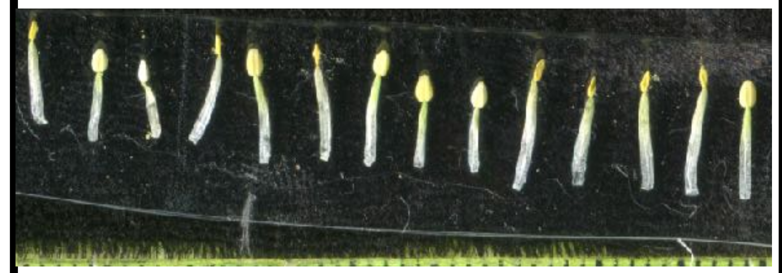	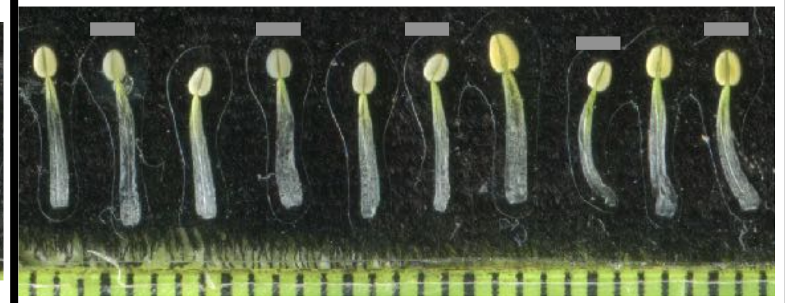	1	1	1	0	0
AjBB4a	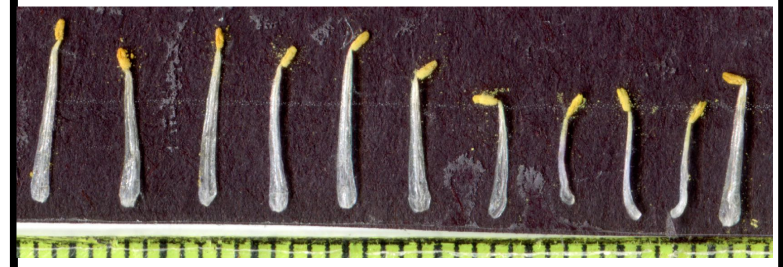	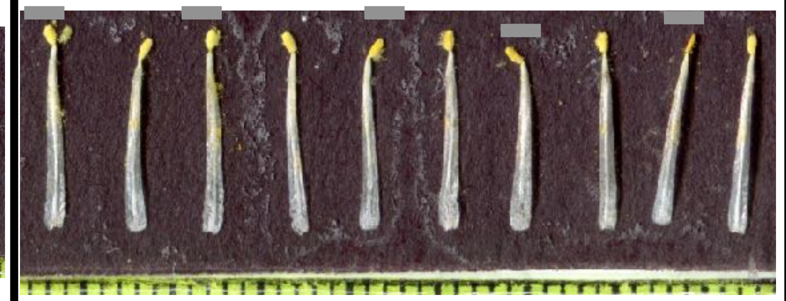	1	1	0	0	0
AjDM4a	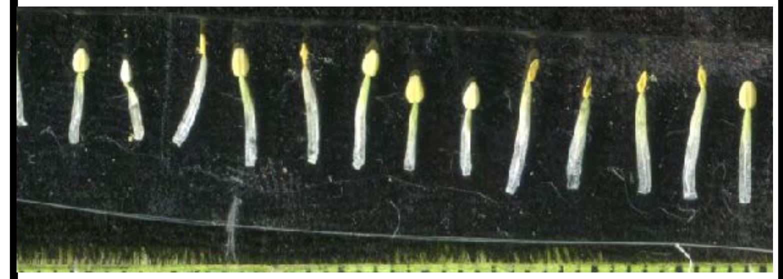	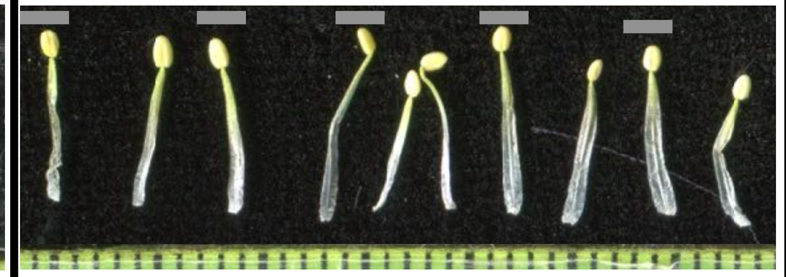	1	1	0	0	0
AjBB3a *from Fig. 1	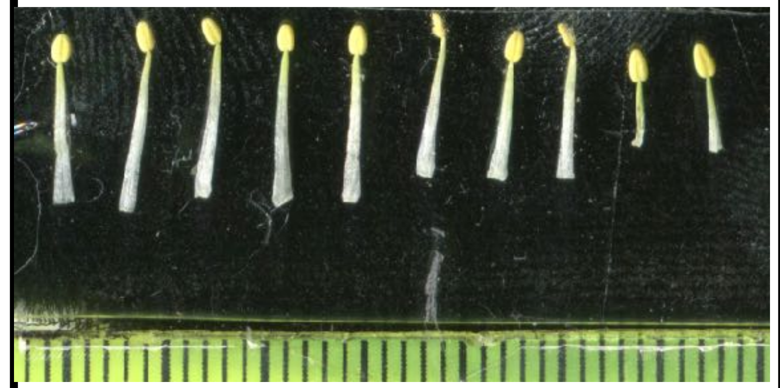	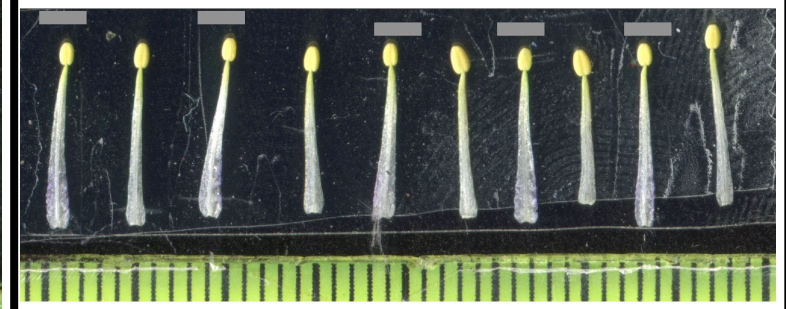	1	1	1	0	0

Figure S2. Variation in stamen morphology of 4 *A. jonesii* flowers. Column 2: scans of dissected organs from the stamen whorls (outside of the two 'staminode' whorls) from inner (L) to outer (R) whorls. Column 3: scans of organs from the inner (grey bars) and outer 'staminode' whorls. Columns 4-8: subtrait scores for each flower. The 4 flowers were dissected from 3 different greenhouse grown plants grown from seed collected from two different populations. See Fig. 2 for scoring scheme.

plant	selection of organs across all stamen whorls	staminode whorls	anther		lateral expansion		fusion
			inner	outer	inner	outer	-
23	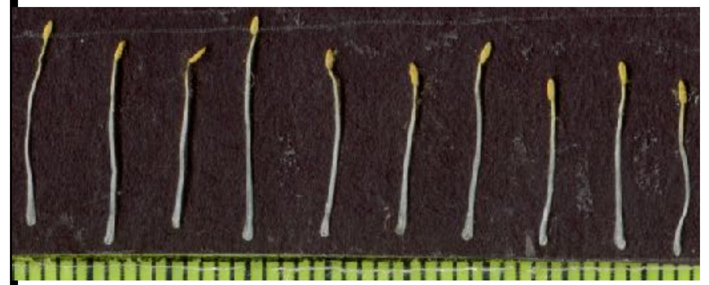	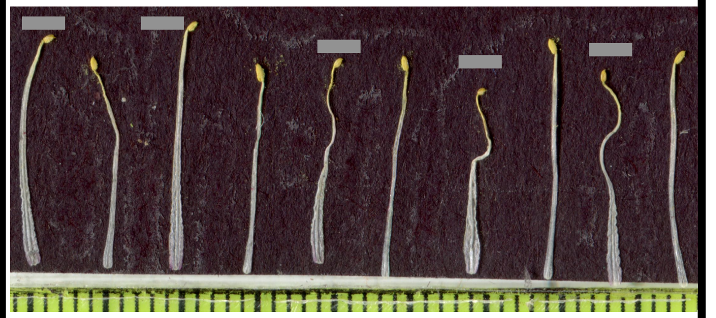	1	1	2	0	0
292	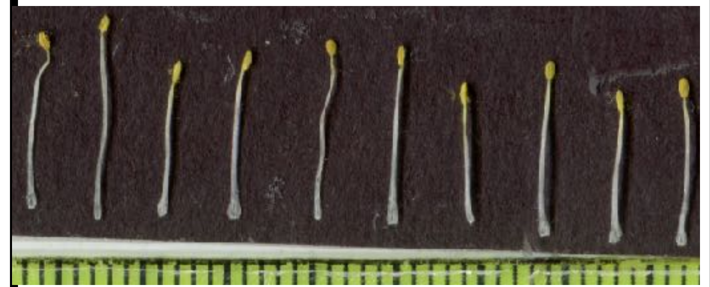	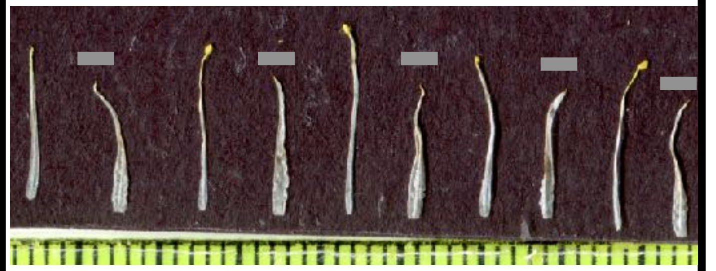	0	1	2	0	0
89	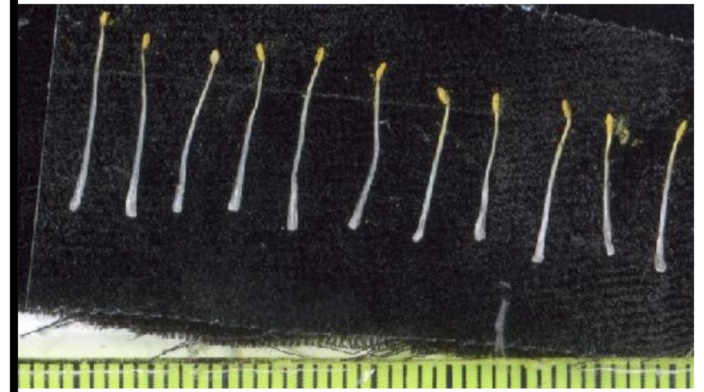	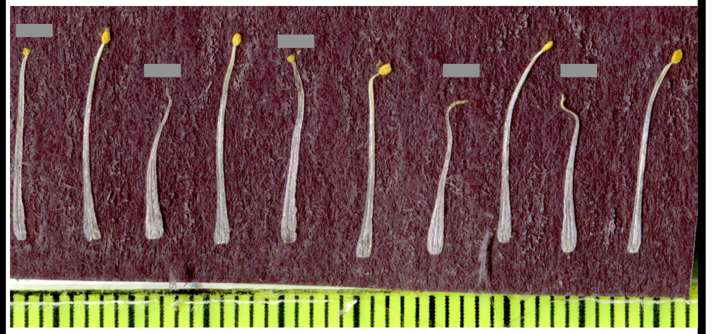	0	1	3	0	0
272	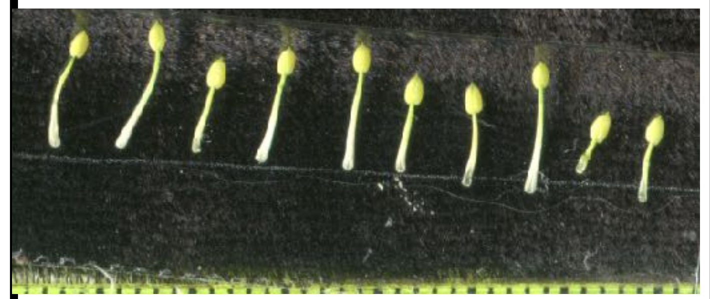	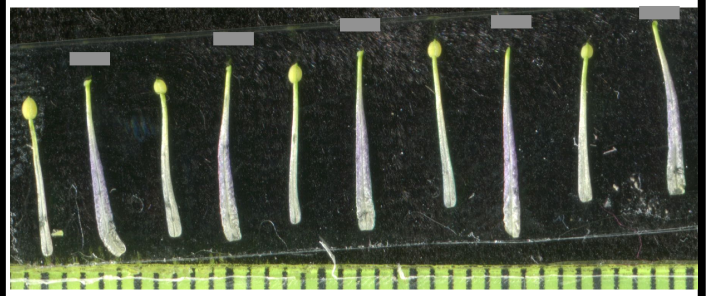	0	1	3	0	0
78	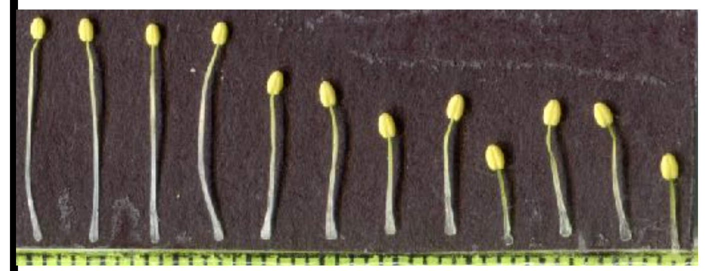	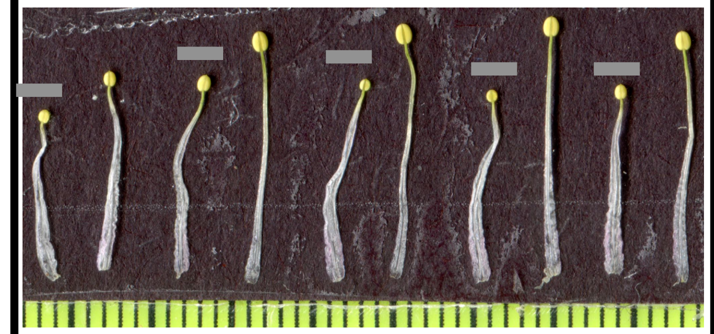	1	1	3	1	0

plant	selection of organs across all stamen whorls	staminode whorls	anther		lateral expansion		fusion
			inner	outer	inner	outer	-
107	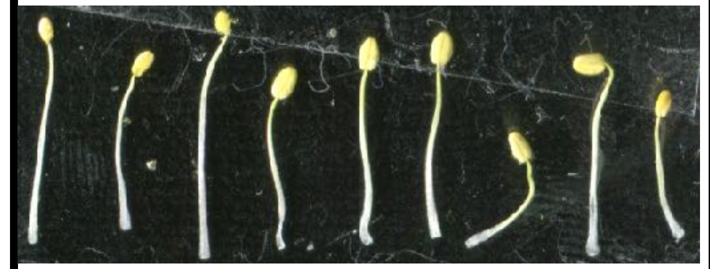	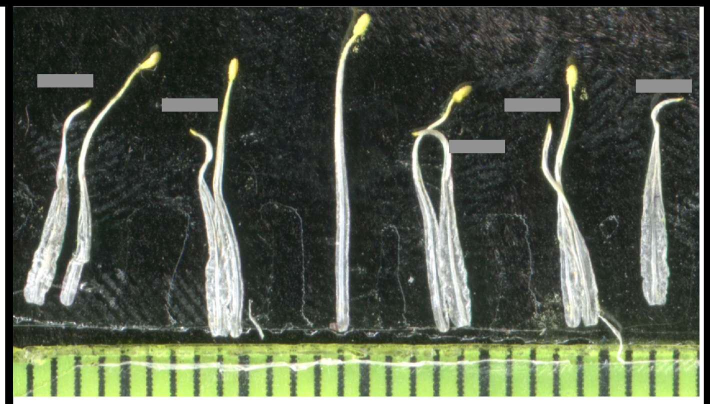	0	1	3	2	1
214	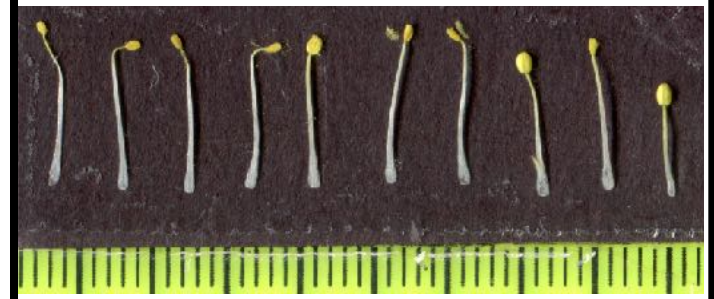	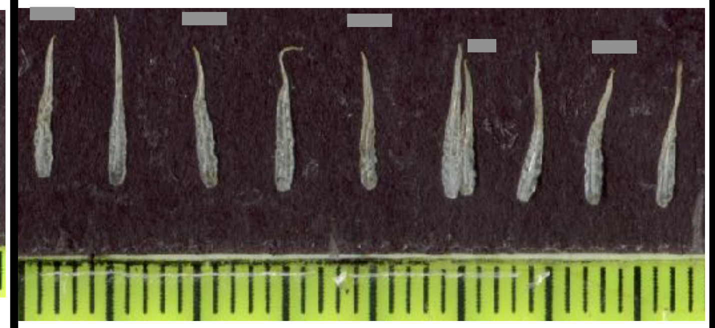	0	0	3	3	0
331	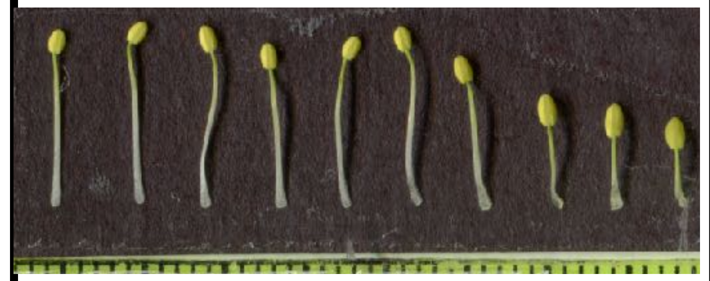	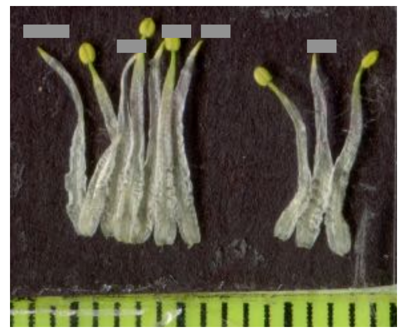	0	1	3	3	1
2	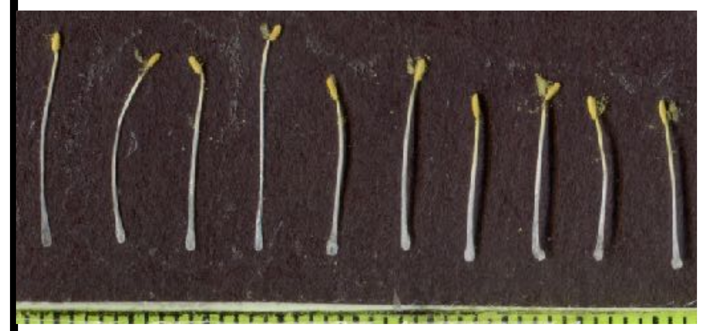	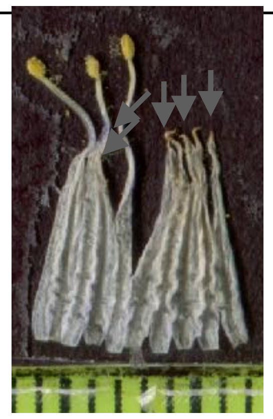	0	1	3	3	1
163	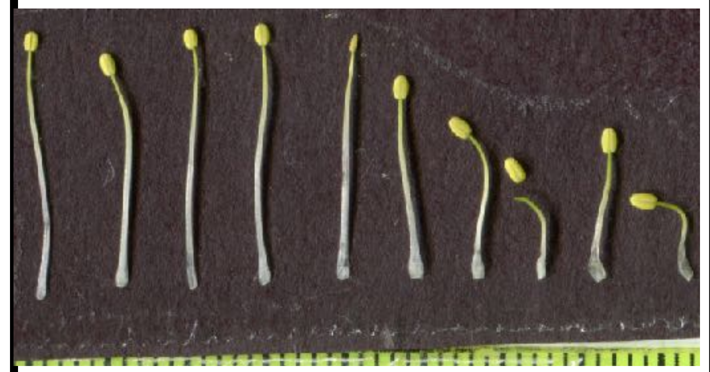	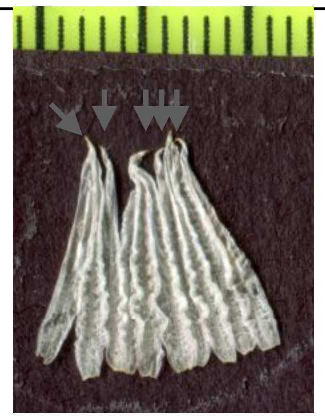	0	0	3	3	1

Figure S3. Variation in stamen and inner and outer 'staminonde' morphology in F2 plants. Column 2: Scans of dissected organs from the stamen whorls (outside of the 'staminode' whorls) from inner (L) to outer (R) whorls. Column 3: Scans of organs from the inner (grey bars/arrows) and outer 'staminode' whorls. Columns 4-8: subtrait scores for the inner two whorls of each flower. See Fig. 2 for details of scoring. Scale: each ruler tick is 1 mm.

plant	histological section	anther		lateral expansion		fusion
		inner	outer	inner	outer	-
237	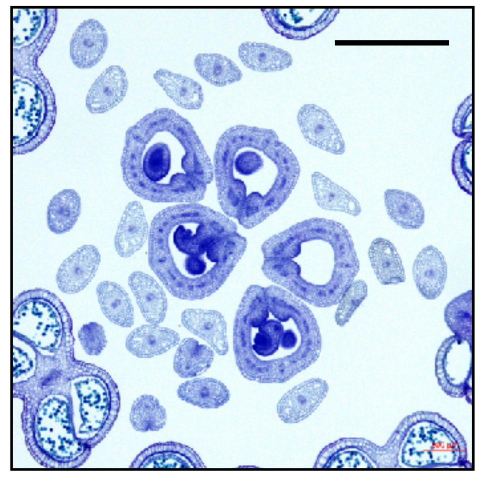	1	1	0	0	0
51	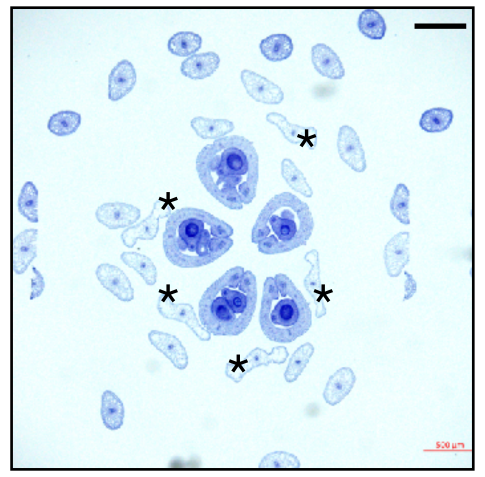	1	1	1	0	0
8	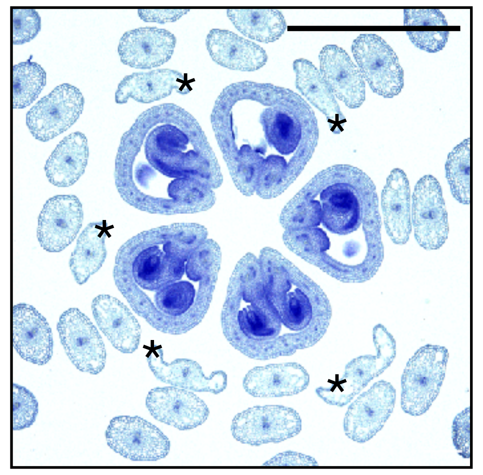	0	1	3	2	1
68	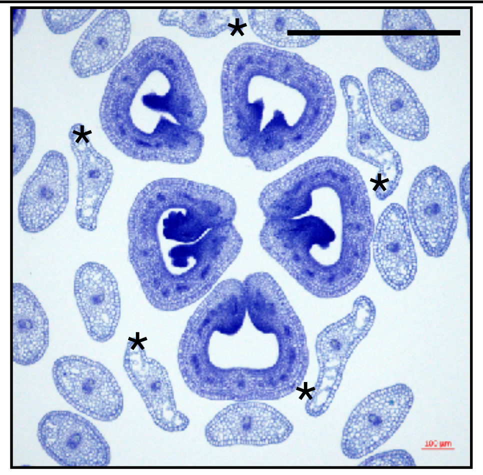	1	1	1	0	0
32	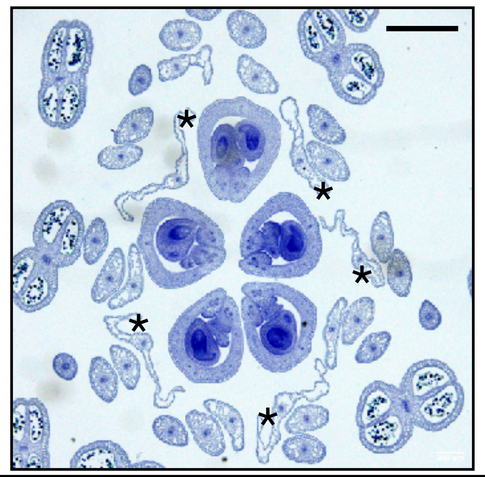	1	1	2	0	0

plant	histological section	anther		lateral expansion		fusion
		inner	outer	inner	outer	-
125	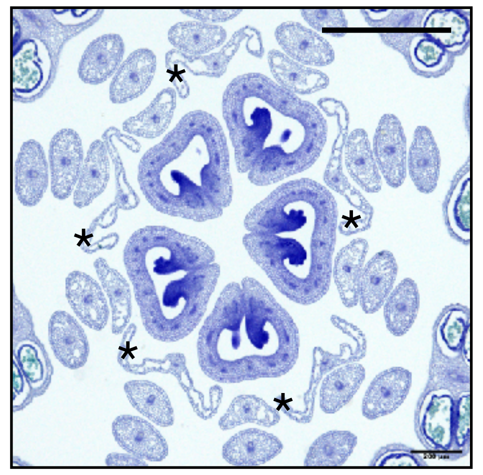	0	1	3	1	0
117	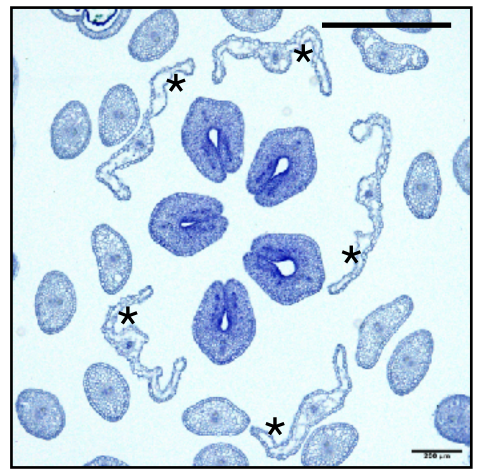	0	1	1	1	0
41	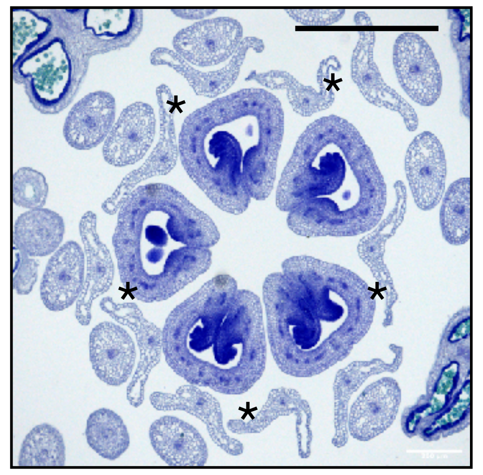	0	0	2	1	0
130	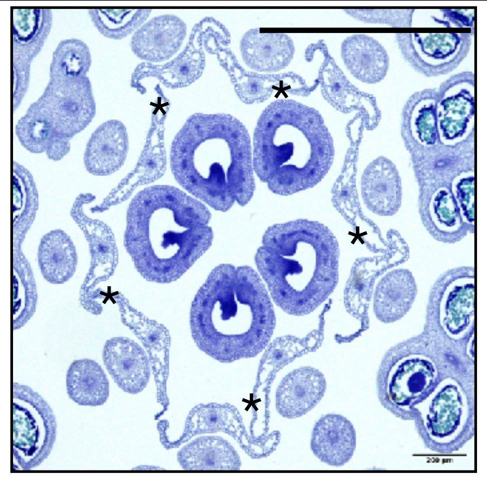	1	1	2	1	1
44	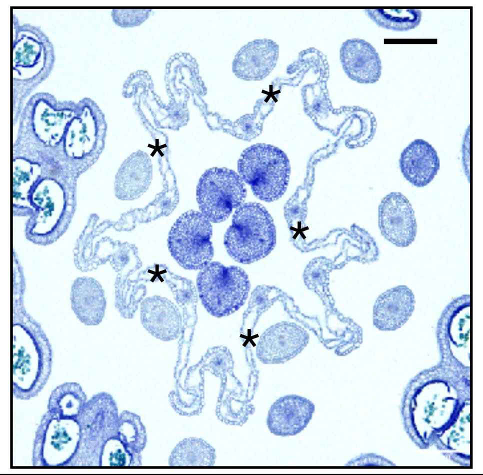	0	0	3	3	1

Figure S4. F2 toluidine blue-stained transverse histological sections from pre-anthesis flower buds. The inner whorl organs are antepetalous (between carpels) and marked with an asterisk where distinguishable. Subtrait scores for the inner two whorls of each flower. See Fig. 2 for details of scoring. Scale bar = 500µm.

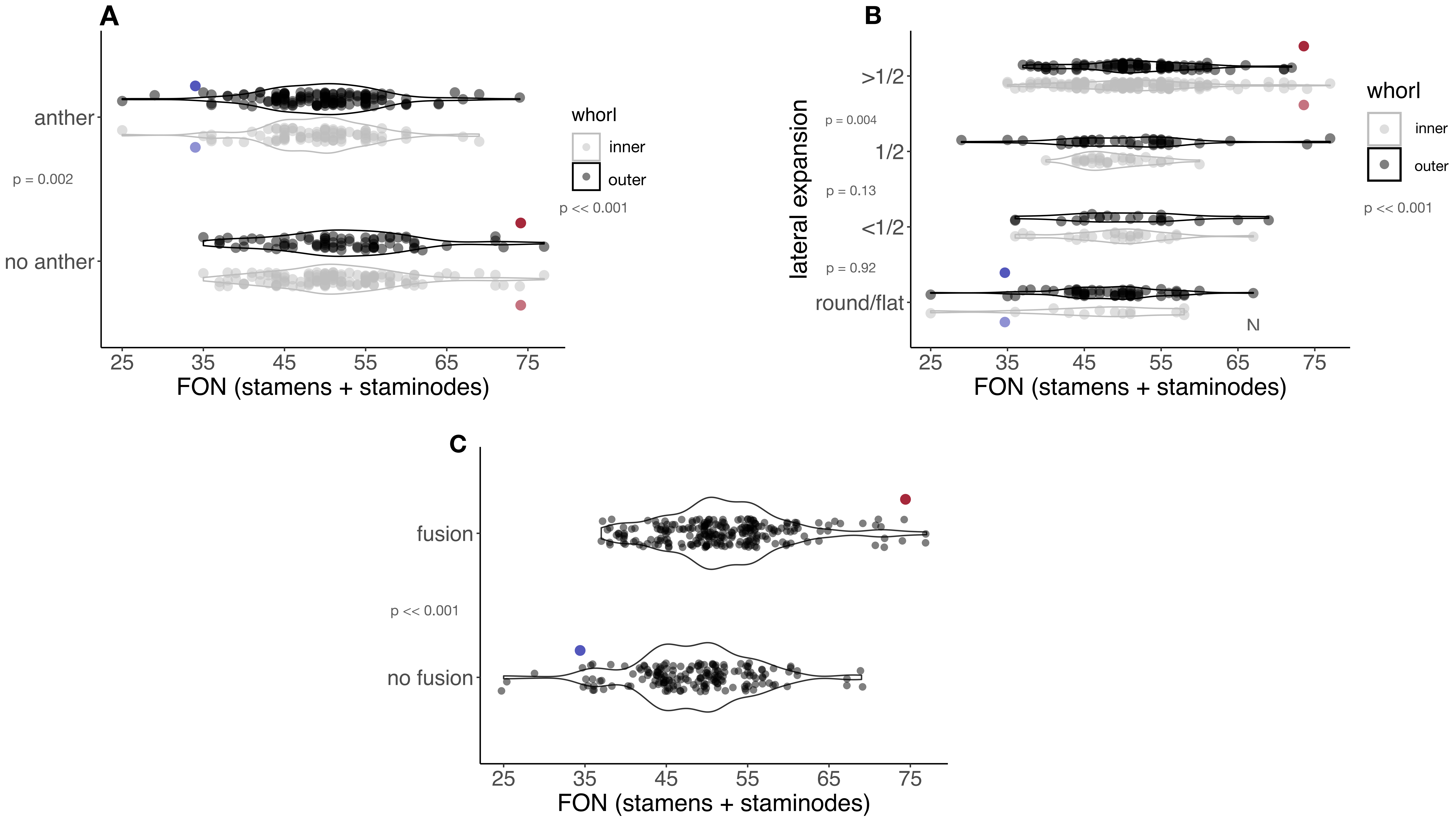

Figure S5. Violin plots of subtrait values predicted by FON (stamens + staminodes) and whorl across F2 flowers for anther (A), lateral expansion (B) and fusion (C) subtraits. We randomly chose 1 flower per plant for plants with two flowers measured to maintain independence of each measurement. 21/231 plants were excluded, as total stamens were not counted for them (n = 210). Mean values are plotted for the parents: *A. jonesii*, blue (n = 6); 'Origami', red (n = 2). Points are jittered to illustrate the distribution, with more jittering for the parents. p-values between LE scores are associated with the transition from the score beneath it to the score above it.

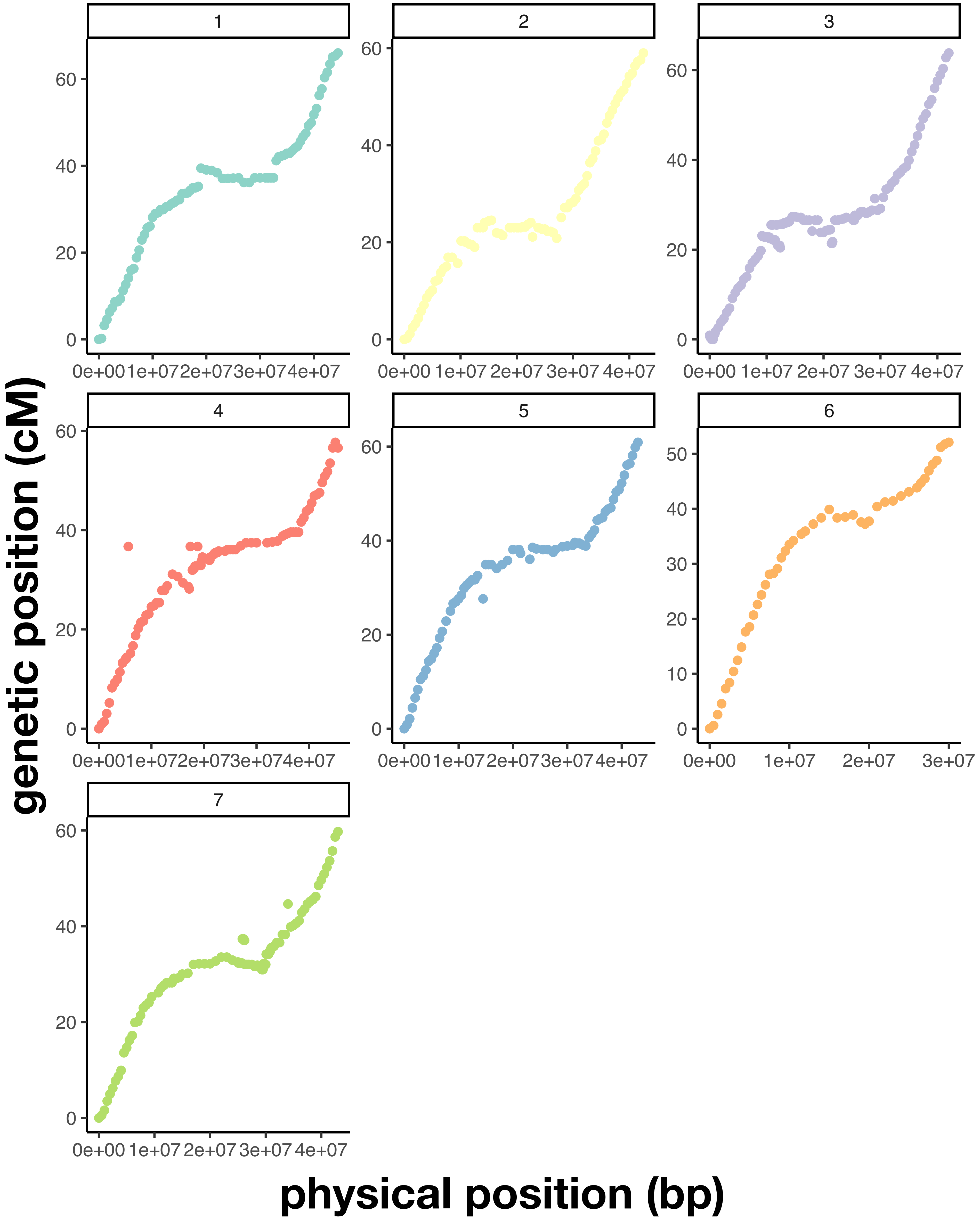

Figure S6. Genetic position (cM) by physical position (bp) across the 7 *Aquilegia* chromosomes. Points represent markers used in genetic map construction.

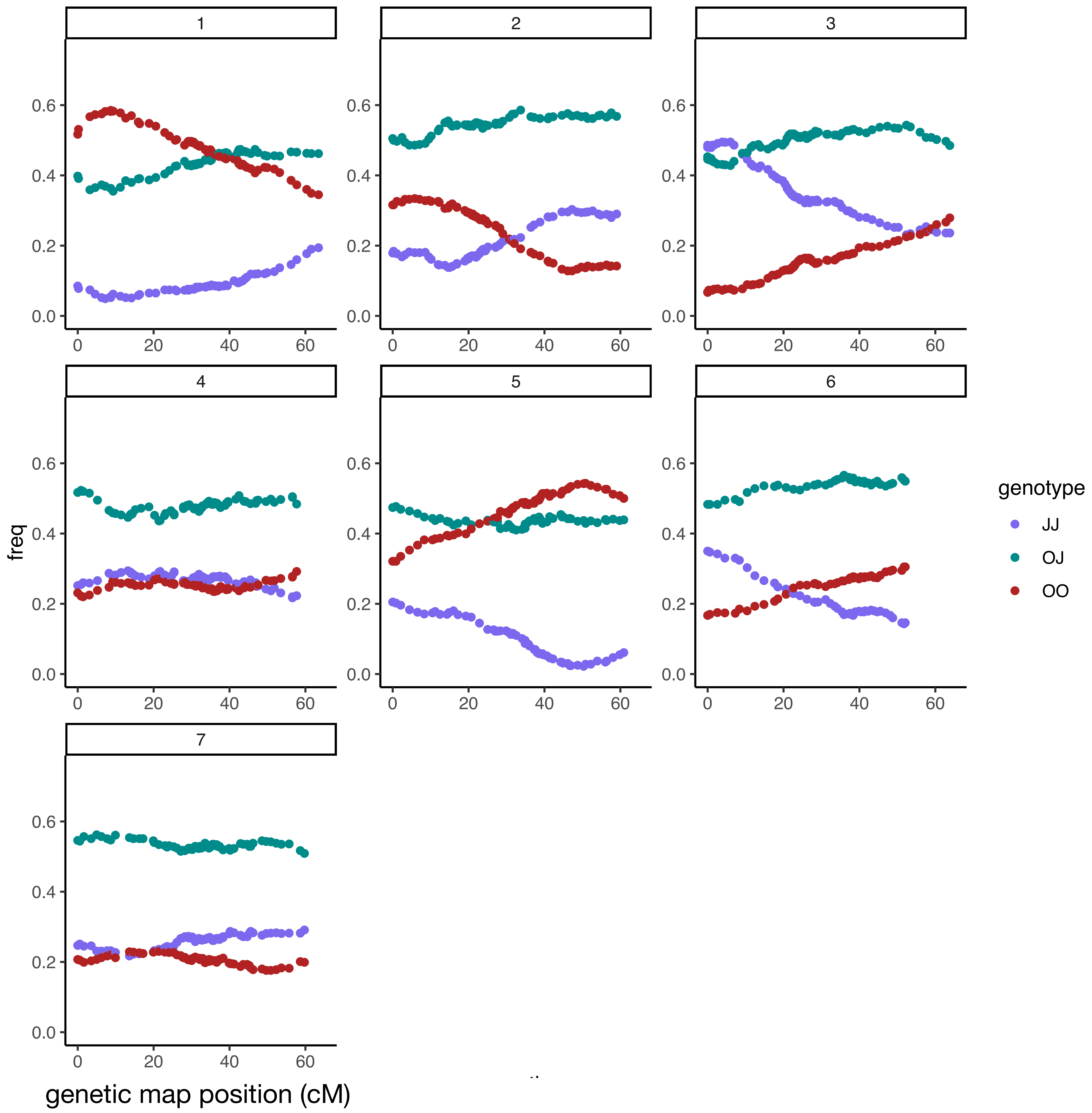

Figure S7. Genotype frequencies in the F2 population across the 7 Aquilegia chromosomes. J, *A. jonesii* allele; O, ‘Origami’ allele. Points represent markers used in genetic map construction.

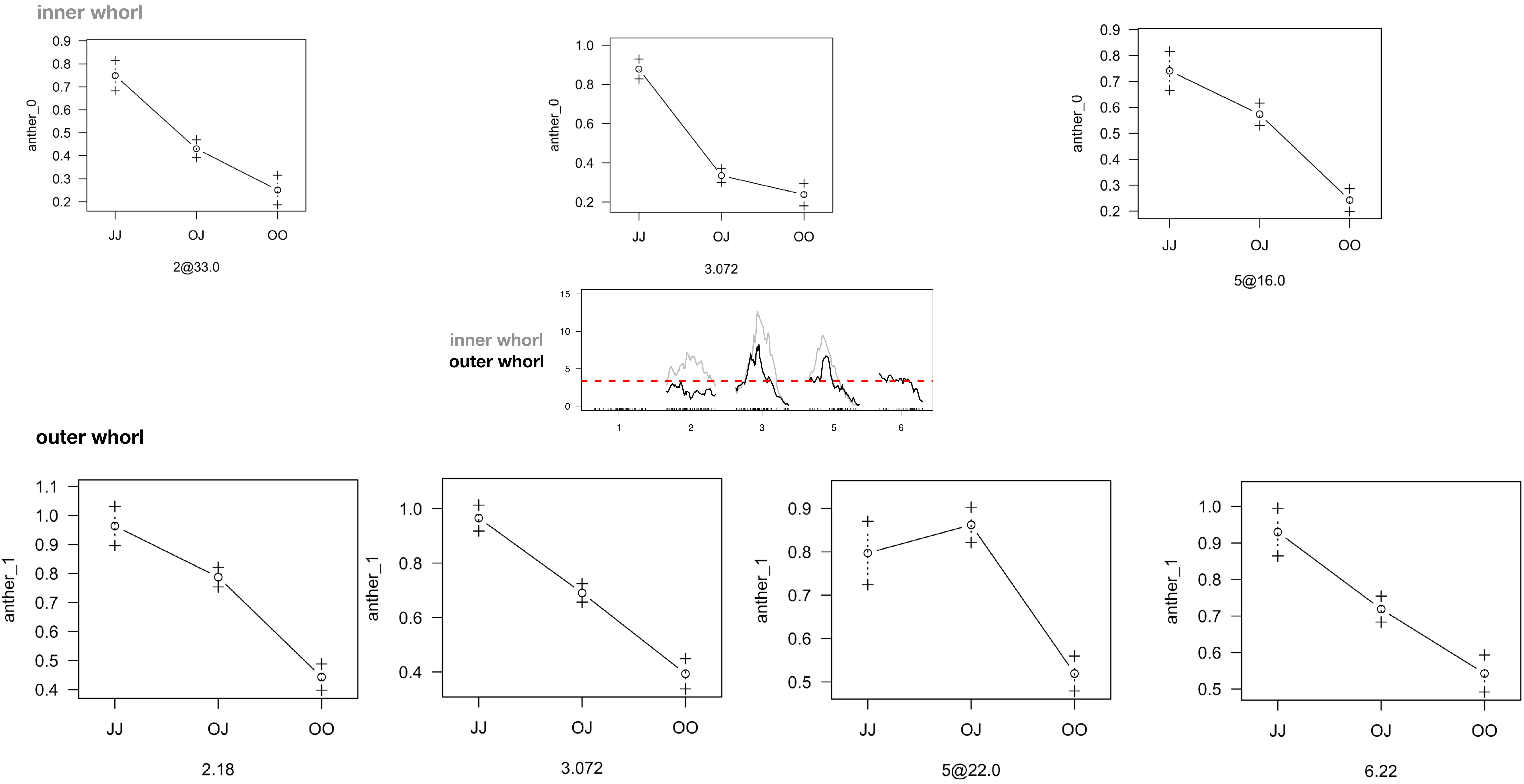

Figure S8. Phenotype by genotype (PxG) effect plots for anther (AN) related QTL. Genotypes are on the x-axis (J = *A. jonesii*, O = ‘Origami’) and their effects are on the y-axis. The chromosome and marker location of each QTL is listed beneath each graph. QTL map included for reference.

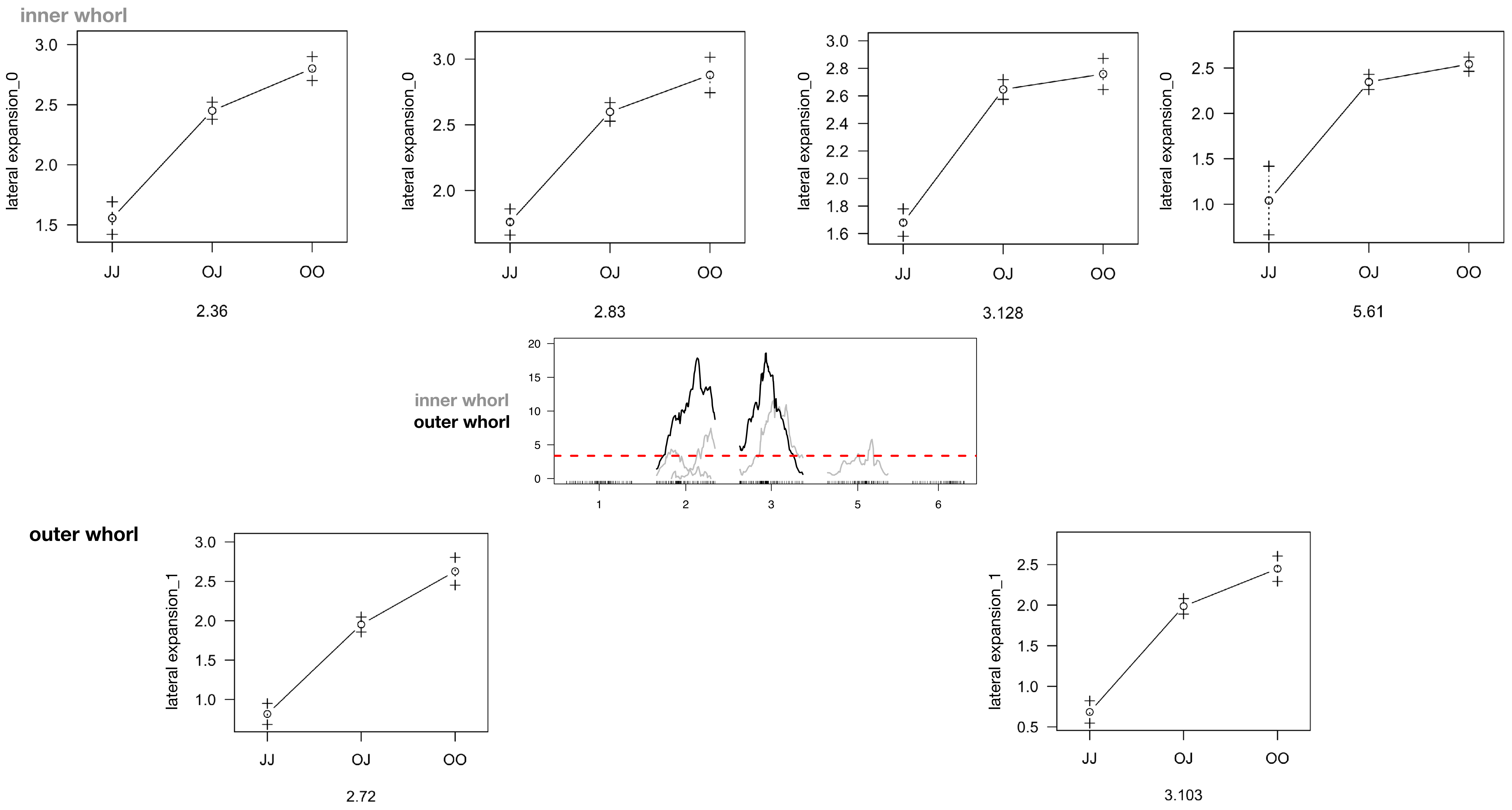

Figure S9. Phenotype by genotype (PxG) effect plots for lateral expansion (LE) related QTL. Genotypes are on the x-axis (J = *A. jonesii*, O = 'Origami') and their effects are on the y-axis. The chromosome and marker location of each QTL is listed beneath each graph. QTL map included for reference.

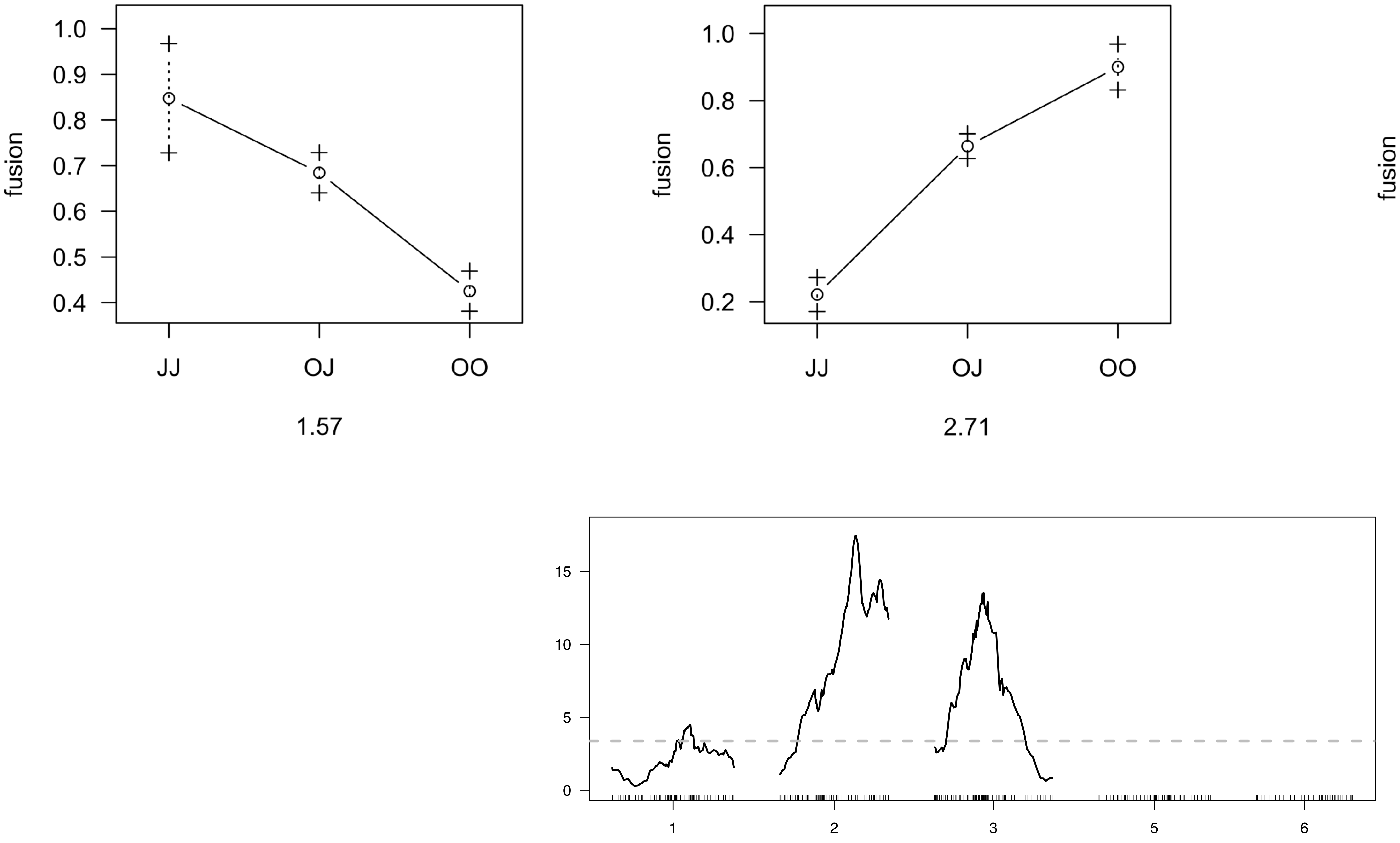

Figure S10. Phenotype by genotype (PxG) effect plots for fusion (FU) related QTL. Genotypes are on the x-axis (J = *A. jonesii*, O = 'Origami') and their effects are on the y-axis. The chromosome and marker location of each QTL is listed beneath each graph. QTL map included for reference.
