# Supplementary material for: Loss of staminodes in Aquilegia jonesii reveals a fading stamen–staminode boundary
Source: EvoDevo. 2024 May 25;15:6. doi: 10.1186/s13227-024-00225-3 (PMC11127400; doi:10.1186/s13227-024-00225-3)
Supplement: Supplementary file 2 — Supplementary Material 2. Contains 8 .pdf tables supplemental to the results of the main text. [file 13227_2024_225_MOESM2_ESM.pdf]

**scores (flw1, flw2)**	**diff**	**inner**	**outer**
0, 0	0	77 (0.46)	34 (0.20)
1, 1	0	62 (0.37)	105 (0.62)
**total**		**139 (0.82)**	**139 (0.82)**
0 ,1	1	30 (0.18)	30 (0.18)
**total**		**30 (0.18)**	**30 (0.18)**

Table S1. Comparison of anther (AN) scores for plants with two flowers measured, by whorl. The number of plants with a given pair of scores is given with the proportions of flowers in a given category in parentheses.

scores (flw1, flw2)	diff	inner	outer
0, 0	0	4 (0.02)	36 (0.21)
1, 1	0	15 (0.09)	10 (0.06)
2, 2	0	17 (0.10)	12 (0.07)
3, 3	0	103 (0.61)	65 (0.38)
**total**		**139 (0.82)**	**123 (0.73)**
0 ,1	1	6 (0.04)	10 (0.06)
1, 2	1	8 (0.05)	11 (0.07)
2, 3	1	12 (0.07)	17 (0.10)
**total**		**26 (0.15)**	**38 (0.22)**
0, 2	2	0 (0.00)	2 (0.01)
1, 3	2	3 (0.02)	3 (0.02)
**total**		**3 (0.02)**	**5 (0.03)**
0, 3	3	1 (0.01)	3 (0.02)
**total**		**1 (0.01)**	**3 (0.02)**

Table S2. Comparison of lateral expansion (LE) scores for plants with two flowers measured, by whorl. The number of plants with a given pair of scores is given with the proportions of flowers in a given category in parentheses.

scores (flw1, flw2)	diff	count
0, 0	0	60 (0.35)
1, 1	0	94 (0.56)
**total**		**154 (0.91)**
0 ,1	1	15 (0.09)
**total**		**15 (0.09)**

Table S3. Comparison of fusion (FU) scores for plants with two flowers measured, by whorl. The number of plants with a given pair of scores is given with the proportions of flowers in a given category in parentheses.

	AN_{OU}	LE_{IN}	LE_{OU}	FU
AN_{IN}	0.9954	-0.7696	-0.6542	-0.6885
	AN_{OU}	-0.5748	-0.6332	-0.6353
		LE_{IN}	0.9205	0.8836
			LE_{OU}	0.9475

Table S4. Matrix of polychoric correlations between subtraits by whorl. "IN" & "OU" refer to the staminode whorls. We randomly chose 1 flower per plant for plants with two flowers measured to maintain independence of each measurement.

		outer whorl		
		presence (1)	absence (0)	total
inner whorl	presence (1)	92 (0.40)	0 (0.00)	92 (0.40)
	absence (0)	65 (0.28)	74 (0.32)	139 (0.60)
	total	157 (0.68)	74 (0.32)	

Table S5. Comparison of anther (AN) scores within flowers between ‘staminode’ whorls. We randomly chose 1 flower per plant for plants with two flowers measured to maintain independence of each measurement. The number of plants with a given pair of scores is given with the proportions of plants in a given category in parentheses.

		outer whorl				
		**none (0)**	**< halfway (1)**	**halfway (2)**	**> halfway (3)**	**total**
inner whorl	**none (0)**	13 (0.06)	0 (0.00)	0 (0.00)	0 (0.00)	**13 (0.06)**
	**< halfway (1)**	26 (0.11)	4 (0.02)	0 (0.00)	0 (0.00)	**30 (0.13)**
	**halfway (2)**	16 (0.07)	10 (0.04)	9 (0.04)	0 (0.00)	**35 (0.15)**
	**> halfway (3)**	11 (0.05)	9 (0.04)	35 (0.15)	98 (0.42)	**153 (0.66)**
		**66 (0.29)**	**23 (0.10)**	**44 (0.19)**	**98 (0.42)**	

Table S6. Comparison of lateral expansion (LE) scores within flowers between ‘staminode’ whorls. We randomly chose 1 flower per plant for plants with two flowers measured to maintain independence of each measurement. The number of plants with a given pair of scores is given with the proportions of plants in a given category in parentheses.

	anther	lateral expansion	fusion
FON (stamens + staminodes)	0.96** (0.93,0.98)	1.05*** (1.02,1.08)	1.07*** (1.04,1.10)
outer whorl	2.83*** (1.9,4.2)	0.35*** (0.24,0.51)	N/A

Table S7. Results of logistic regressions of each subtrait predicted by FON (stamens + staminodes) and whorl. Odds ratios are reported with 95% confidence intervals in parentheses. Asterisks represent significant p-values: $^{**}p < 0.01$, $^{***}p << 0.001$

subtrait	whorl	qtl	aq_locus_name	aq_peptid_name	at_best_hit	at_symbol	arabi-defline	GO
anther	inner/outer	Q2	Aqcoe2G168200	Aqcoe2G168200.1	AT3G18010.1	WOX1	WUSCHEL related homeobox 1	GO:0003677
anther	outer	Q6	Aqcoe6G031900	Aqcoe6G031900.1	AT5G20240.1	PI	K-box region and MADS-box transcription factor family protein	GO:0006355,GO:0005634,GO:000370
anther	outer	Q6	Aqcoe6G257100	AP3-1	AT3G54340.1	AP3,ATAP3	K-box region and MADS-box transcription factor family protein	GO:0006355,GO:0005634,GO:000370
anther	outer	Q3	Aqcoe3G202800	Aqcoe3G202800.1	AT5G63090.1	LOB	Lateral organ boundaries (LOB) domain family protein	
lateral expansion	inner	Q5	Aqcoe5G352300	Aqcoe5G352300.1	AT1G24260.1	AGL9,SEP3	K-box region and MADS-box transcription factor family protein	GO:0046983,GO:0003677,GO:000635
fusion	NA	Q1	Aqcoe1G419900	Aqcoe1G419900.1	AT3G55950.1	ATCRR3,CCR3	CRINKLY4 related 3	GO:0006468,GO:0005524,GO:000467
fusion	NA	Q1	Aqcoe1G308600	Aqcoe1G308600.1	AT2G20810.1	GAUT10,LGT4	galacturonosyltransferase 10	GO:0016757,GO:0047262
fusion	NA	Q1	Aqcoe1G365400	Aqcoe1G365400.1	AT5G54690.1	GAUT12,IRX8,LGT6	galacturonosyltransferase 12	GO:0016757,GO:0047262
fusion	NA	Q1	Aqcoe1G375000	Aqcoe1G375000.1	AT5G47780.1	GAUT4	galacturonosyltransferase 4	GO:0016757,GO:0047262
adhesion	-	none	Aqcoe7G029100	Aqcoe7G029100.1	AT5G23940.1	DCR,EMB3009,PEL3	HXXXD-type acyl-transferase family protein	GO:0016747
adhesion	-	none	Aqcoe6G082400	Aqcoe6G082400.1	AT1G78240.1	OSU1,QUA2,TSD2	S-adenosyl-L-methionine-dependent methyltransferases superfamily protein	GO:0008168
adhesion	-	none	Aqcoe7G043400	Aqcoe7G043400.1	AT4G00740.1	QUA3	S-adenosyl-L-methionine-dependent methyltransferases superfamily protein	GO:0008168
adhesion	-	none	Aqcoe3G134900	Aqcoe3G134900.1	AT3G49720.1	CGR2		
adhesion	-	none	Aqcoe6G316700.1		AT5G65810	CGR3		
adhesion	-	none	Aqcoe1G415600	Aqcoe1G415600.1	AT5G58600.1	PMR5,TBL44	Plant protein of unknown function (DUF828)	
ab/adaxial polarity	-	none	Aqcoe1G459000	Aqcoe1G459000.1	AT2G37630.1	AS1,ATMYB91,ATPHAN,MYB91	myb-like HTH transcriptional regulator family protein	GO:0010338,GO:0006351,GO:000563
ab/adaxial polarity	-	none	Aqcoe2G438700	Aqcoe2G438700.1	AT1G65620.3	AS2	Lateral organ boundaries (LOB) domain family protein	
ab/adaxial polarity	-	none	Aqcoe1G143700	Aqcoe1G143700.1	AT5G60690.1	IFL,IFL1,REV	Homeobox-leucine zipper family protein / lipid-binding START domain-containing	GO:0003677,GO:0008289
ab/adaxial polarity	-	none	Aqcoe1G178700	Aqcoe1G178700.1	AT2G34710.1	ATHB-14,ATHB14,PHB,PHB-1D	Homeobox-leucine zipper family protein / lipid-binding START domain-containing	GO:0003677,GO:0008289
ab/adaxial polarity	-	none			AT2G46685	miR166/ATBH15		
ab/adaxial polarity	-	none	Aqcoe1G248400	Aqcoe1G248400.2	AT2G33860.1	ARF3,ETT	Transcriptional factor B3 family protein / auxin-responsive factor AUX/IAA-related	GO:0003677,GO:0009725,GO:000635
ab/adaxial polarity	-	none	Aqcoe1G248400	Aqcoe1G248400.2	AT2G33860.1	ARF3,ETT	Transcriptional factor B3 family protein / auxin-responsive factor AUX/IAA-related	GO:0003677,GO:0009725,GO:000635
ab/adaxial polarity	-	none	Aqcoe1G074300.1		AT2G45190	FILAMENTOUS FLOWER,YABBY1		
ab/adaxial polarity	-	none	Aqcoe1G074300.1		AT1G08465	YABBY2		
ab/adaxial polarity	-	none	Aqcoe7G386400.1		AT2G26580	YABBY5		
ab/adaxial polarity	-	none	Aqcoe3G065300	Aqcoe3G065300.1	AT1G69180.1	CRC	Plant-specific transcription factor YABBY family protein	GO:0007275
ab/adaxial polarity	-	none	Aqcoe3G410900	Aqcoe3G410900.1	AT5G16560.1	KAN,KAN1	Homeodomain-like superfamily protein	
ab/adaxial polarity	-	none	Aqcoe3G169700	Aqcoe3G169700.1	AT1G32240.1	KAN2	Homeodomain-like superfamily protein	
antagonistsic with AG	-	none	Aqcoe5G437300	Aqcoe5G437300.1	AT5G41410.1	BEL 1	POX (plant homeobox) family protein	GO:0006355,GO:0003677
antagonistsic with AG	-	none	Aqcoe2G233200	Aqcoe2G233200.1	AT5G02030.1	BLH9,BLR,HB-6,LSN,PNY,RPL,VAN	POX (plant homeobox) family protein	GO:0006355,GO:0003677
antagonistsic with AG	-	none	Aqcoe1G138800	Aqcoe1G138800.1	AT2G27990.1	BLH8,PNF	BEL1-like homeodomain 8	GO:0006355,GO:0003677
antagonistsic with AG	-	none	Aqcoe1G341500	Aqcoe1G341500.1	AT1G62360.1	BUM,BUM1,SHL,STM,WAM,WAM1	KNOX/ELK homeobox transcription factor	GO:0003677,GO:0006355,GO:000563
antagonistsic with AG	-	none	Aqcoe3G280300.1		AT1G70510	KNAT2		
anther development	-	none	Aqcoe4G024000	Aqcoe4G024000.1	AT4G18960.1	AG	K-box region and MADS-box transcription factor family protein	GO:0046983,GO:0003677,GO:000635
anther development	-	none	Aqcoe7G042600	Aqcoe7G042600.1	AT5G07280.1	EMS1,EXS	Leucine-rich repeat transmembrane protein kinase	GO:0005515,GO:0006468,GO:000552
anther development	-	none	Aqcoe1G368600	Aqcoe1G368600.1	AT4G21330.1	DYT1	basic helix-loop-helix (bHLH) DNA-binding superfamily protein	
anther development	-	none	Aqcoe3G100100	Aqcoe3G100100.1	AT3G28470.1	ATMYB35,TDF1	Duplicated homeodomain-like superfamily protein	
anther development	-	none	Aqcoe3G122900	Aqcoe3G122900.1	AT2G16910.1	AMS	basic helix-loop-helix (bHLH) DNA-binding superfamily protein	GO:0046983
anther development	-	none	Aqcoe1G380400	Aqcoe1G380400.1	AT5G56110.1	AtMYB103,ATMYB80,MS188,MYB10	myb domain protein 103	
anther development	-	none	Aqcoe2G003200	Aqcoe2G003200.1	AT3G11440.1	ATMYB65,MYB65	myb domain protein 65	
anther development	-	none	Aqcoe1G085500	Aqcoe1G085500.1	AT3G09090.1	DEX1	defective in exine formation protein (DEX1)	
anther development	-	none	Aqcoe3G100100	Aqcoe3G100100.1	AT3G28470.1	ATMYB35,TDF1	Duplicated homeodomain-like superfamily protein	
anther development	-	none	Aqcoe3G283200	Aqcoe3G283200.1	AT1G23420.1	INO	Plant-specific transcription factor YABBY family protein	GO:0007275
anther development	-	none	Aqcoe5G346200	Aqcoe5G346200.1	AT4G24972.1	TPD1	tapetum determinant 1	
anther development	-	none	Aqcoe7G363100	Aqcoe7G363100.1	AT2G13680.1	ATGSL02,CALS5,GLS2	callose synthase 5	GO:0016020,GO:0006075,GO:000384
anther development	-	none	Aqcoe3G159700	Aqcoe3G159700.1	AT5G14070.1	ROXY2	Thioredoxin superfamily protein	GO:0045454,GO:0015035,GO:000905
anther development	-	none	no protein homolog matches in Aquilegia		AT4G27330	NZZ/SPL		
anther development	-	none	Aqcoe4G227800	Aqcoe4G227800.1	AT4G20090.1	EMB1025	Pentatricopeptide repeat (PPR) superfamily protein	
anther development	-	none	Aqcoe7G083800	Aqcoe7G083800.1	AT4G37590.1	NPY5	Phototropic-responsive NPH3 family protein	
B-class transcription	-	none	Aqcoe6G257300	AP3-2	AT3G54340.1	AP3,ATAP3	K-box region and MADS-box transcription factor family protein	GO:0006355,GO:0005634,GO:000370
floral meristem ID	-	none	Aqcoe0095s0001	Aqcoe0095s0001.1	AT1G30950.1	UFO	F-box family protein	GO:0005515
floral meristem ID	-	none	Aqcoe1G456700	Aqcoe1G456700.1	AT5G03680.1	PTL	Duplicated homeodomain-like superfamily protein	
floral meristem ID	-	none	Aqcoe5G327800	Aqcoe5G327800.1	AT5G61850.1	LFY,LFY3	floral meristem identity control protein LEAFY (LFY)	GO:0006355,GO:0003677

Table S8. Homologs of candidate genes searched for within and across subtraits
